# Epidemiology of multimorbidity associated with atherosclerotic cardiovascular disease in the United States, 1999–2018

**DOI:** 10.1186/s12889-023-17619-y

**Published:** 2024-01-23

**Authors:** Ying Tian, Dongna Li, Haoliang Cui, Xin Zhang, Xiaoyan Fan, Feng Lu

**Affiliations:** 1 Clinical Research Center, Affiliated Hospital of Shandong University of Traditional Chinese Medicine, Jinan, Shandong China; 2 Department of Cardiology, Affiliated Hospital of Shandong University of Traditional Chinese Medicine, Jinan, Shandong China; 3 School of Public Health, Peking University, Beijing, 100191 China

**Keywords:** Atherosclerotic cardiovascular disease, Multimorbidity, National Health and Nutrition Examination Survey

## Abstract

**Background:**

The multimorbidity of Atherosclerotic cardiovascular disease (ASCVD) and many other chronic conditions is becoming common. This study aimed to assess multimorbidity distribution in ASCVD among adults in the United States from 1999 to 2018.

**Methods:**

This cross-sectional survey from the National Health and Nutrition Examination Survey (NHANES) 1999–2018 using stratified multistage probability design. Among the 53,083 survey respondents during the study period, 5,729 US adults aged ≥ 20 years with ASCVD. Joinpoint regression was used to assess the statistical significance of prevalence trends in the prevalence of ASCVD stratified by multimorbidity. The Apriori association rule mining algorithm was used to identify common multimorbidity association patterns in ASCVD patients.

**Results:**

Overall, 5,729 of 53,083 individuals had ASCVD, and the prevalence showed a slow declining trend (biannual percentage change = -0.81%, p = 0.035, average 7.71%). The prevalence of ASCVD significantly decreased in populations without dyslipidemia, diabetes mellitus (DM), hypertension, asthma, chronic obstructive pulmonary disease (COPD), and arthritis (all groups, *p* < 0.05). Additionally, 65.6% of ASCVD patients had at least four of the 12 selected chronic conditions, with four and five being the most common numbers of conditions (17.9% and 17.7%, respectively). The five most common chronic conditions were (in order) dyslipidemia, hypertension, arthritis, chronic kidney disease, and DM. The coexistence of hypertension and dyslipidemia had the highest support in association rules (support = 0.63), while the coexistence of dyslipidemia, hypertension, metabolic syndrome, and DM had the highest lift (lift = 1.82).

**Conclusions:**

During the 20-year survey period, there was a significant decrease in the overall prevalence of ASCVD. However, this reduction was primarily observed in individuals without dyslipidemia, DM, hypertension, asthma, COPD, and arthritis. Among populations with any of the evaluated chronic conditions, the prevalence of ASCVD remained unchanged. Most of ASCVD patients had four or more concurrent chronic conditions.

**Supplementary Information:**

The online version contains supplementary material available at 10.1186/s12889-023-17619-y.

## Background

Atherosclerotic cardiovascular disease (ASCVD) is the most common cause of death worldwide, with 460,000 deaths occurring over 7 years, accounting for one-third of all deaths [1]. In 2019, heart disease ranked first among the top 10 causes of death in the United States [2]. Among the important risk factors for ASCVD, control rates of hyperlipidemia and hypertension as well as the patient's medication adherence remain suboptimal, and the prevalence of diabetes mellitus is increasing dramatically, all of which limit the effectiveness of primary prevention of ASCVD [3–6]. What makes the situation even more complex is the aging of the global population. Population aging has accentuated multimorbidity in ASCVD patients, complicated medication requirement, made treatment more difficult, and even increased mortality [7, 8]. Therefore, the prevention and therapeutic of ASCVD remains highly challenging.

The World Health Organization defines multimorbidity as a condition in which two or more chronic conditions occur simultaneously, based on epidemiological and public health perspectives [9]. These conditions are in an individual. The multimorbidity of ASCVD and many other chronic conditions is becoming common. Many chronic diseases share risk factors as well as biological pathways. Therefore, their prevention and treatment also have important intersections. There may be correlations between ASCVD and some of these conditions; for example, some may be aggregated by ASCVD. Judging by the prognosis, multimorbidity lead to a worse prognosis for patients; they are more likely to experience premature death, hospital admissions, and extended hospital stays than those with a single disease [10]. Therefore, patients may benefit from a focus on multimorbidity with comprehensive consideration of these diseases, active prevention, and rational medication use.

The Department of Health and Human Services and the Institute of Medicine convened a stakeholder meeting that included the American Heart Association and American College of Cardiology (ACC) to identify core principles for clinical practice guidelines in the effective management of people with multiple chronic conditions in 2012 [11]. Eleven principles were identified during the meeting to improve the attentiveness of the guidelines for those with multiple chronic conditions [12]. The ACC developed an expert consensus on the decision pathway for integrating ASCVD and multimorbidity treatment in 2022 [7]. Special attention was given to delaying disease progression and reducing the risk of adverse events, particularly in the context of multiple comorbidities.

Previous studies have already investigated some chronic conditions associated with ASCVD. Epidemiology of multimorbidity in older adults with cardiovascular disease [13], the multimorbidity rate and common chronic conditions of cardiovascular disease in China among people aged 45 years or older [14], the relationship between comorbidities and health-related quality of life in Slovenian patients with coronary heart disease (CHD) [15], the relationship between the burden of comorbidities and the social determinants of health in patients with ASCVD in the United States [16], the prevalence of cardiac-renal-metabolic (CRM) comorbidities [17], common non-cardiovascular multimorbidity patterns and their association with clinical outcomes [18], etc. It is evident that the multimorbidity of ASCVD have been gradually emphasized. However, no studies have been done on the changes in the prevalence trends of ASCVD over the past 20 years according to multimorbidity stratification and the associations among the common co-morbidities of ASCVD. Therefore, this study was designed.

## Methods

### Data source

The National Health and Nutrition Examination Survey (NHANES) is a series of cross-sectional, nationally representative, noninstitutional US population surveys designed to assess the health and nutritional status of the US population. These surveys are completed through face-to-face interviews and health screenings at mobile screening centers, using a complex, stratified, multistage probability cluster sampling design [19].

The current analysis was performed using data from 1999–2000 to 2017–2018 for adults aged ≥ 20 years. This study was approved by the Institutional Review Board of the National Center for Health Statistics, and all participants provided written informed consent. The analyses presented here needed no further ethical approval.

### Data collection

Information collected included age, sex, race/ethnicity (non-Hispanic white, non-Hispanic black, Hispanic, or others), education (less than high school, high school graduate, or above high school), family income-to-poverty ratio (< 1.3, 1.3–3.49, or ≥ 3.5), health insurance status (uninsured or insured), and smoking status (non-smoker, former smoker, or current smoker). ASCVD and 12 other selected chronic conditions were: chronic liver disease (CLD), arthritis, chronic kidney disease (CKD), asthma, chronic obstructive pulmonary disease (COPD), diabetes mellitus (DM), dyslipidemia, hypertension, metabolic syndrome (MetS), congestive heart failure (CHF), cancer and obesity.

### Diagnostic criteria for ASCVD and multimorbidity

The ASCVD diagnosis was based on self-reported medical history and cardiovascular drug use, including those related to coronary heart disease (questionnaire: mcq160c), angina/angina pectoris (mcq160d), heart attack/myocardial infarction (mcq160e), and stroke (mcq160f) [20].

We defined 12 diseases associated or potentially associated with ASCVD from the list of chronic conditions [21], including arthritis, cancer, CKD, DM, dyslipidemia, hypertension, MetS, CHF, CLD, asthma, COPD, and obesity. These diseases were prevalent and were limited by the cross-sectional survey of the NHANES database, which did not allow for a clear sequence of occurrence between diseases. The diagnostic criteria for the above diseases are described in Appendix 1**(**Supplement).

### Outcomes

The primary outcomes were the distribution of multimorbidity in ASCVD and the association rules between multimorbidities. The secondary outcome was the correlation between ASCVD and multimorbidity.

### Statistical analysis

Using data from 10 2-year cycles (1999–2000 to 2017–2018), the study analyzed the distribution of ASCVD patients among the 12 chronic-condition populations, as well as the overall prevalence trends of ASCVD stratified by those conditions.

The Joinpoint regression model was used to identify mean biannual percentage changes (BPC) and Average Biannual Percent Change (ABPC) in trends for the age-adjusted estimated proportion among all adults with multimorbidity in ASCVD [22]. Joinpoint regression was used to assess the statistical significance of the trends.

In the visualized association rule analysis, the Apriori association rule mining algorithm was used to identify common multimorbidity association patterns in ASCVD patients using three measures of association rules [23]: (1) support: indicating the probability of multimorbidity of disease A and disease B; (2) confidence: indicating the probability of multimorbidity of disease B among those with disease A; (3) lift: indicating the ratio of the actual multimorbidity of disease A and disease B to the expected multimorbidity. Greater lift values (> 1) indicated stronger associations. In order to generate the association rules, the modeling process set the minimum support degree at 0.1, minimum confidence degree at 0.6, minimum lift degree at 1, and minimum number of terms to 2. After obtaining the association rules, we visualized and analyzed them for better observation of the associations.

For the secondary outcome, weighted univariate and multivariate binary logistic regression analyses based on generalized linear models were used to explore the risk factors associated with ASCVD. Independent variables were selected using the stepwise regression method. The Akaike information criterion (AIC) was utilized as the judgment criteria, and the minimum AIC information statistics were selected to obtain the overall optimal model [24]. We used odds ratios as a measure of effect. Multivariate logistic regression including product terms was used to explore the possible multiplicative scale interactions between variables (model adjusted for age, sex, race/ethnicity, education, Ratio of family income to poverty and smoking status). Furthermore, on an additive scale, we quantified the interaction or joint effect of two factors by calculating the risk that was more than expected based on the independent effects of these factors. A 2 * 2 addition model was constructed for statistically significant interaction terms to determine whether the interaction terms had an additive interaction from a biological perspective. Interaction was determined by calculating the relative excess risk due to interaction (RERI), attributable proportion due to interaction(AP), and synergy index(S). When the CI of RERI and AP do not contained 0 and the CI of S does not contained 1, there was additive interaction.

Missing data of outcome variables in ASCVD and of other variables (< 1%) were removed. Missing data for continuous variables were subjected to multiple imputation with predicted mean matching, while missing data for categorical variables were used as new categories. All statistical analyses used the imputed dataset.

To obtain the nationally representative estimates of the US, all analyses used weighted samples considering the stratification and clustering of the design and sampling weights estimated as the inverse probability of being selected for the survey [25]. To provide estimates for the entire 20 years, a weight variable sample was created by taking two-tenths of the 4-year weight for each person sampled in 1999–2002 and one-tenth of the 2-year weight for each person sampled in 2003–2018.

All statistical analyses were performed using R Statistical Software (v4.2.0, R Foundation for Statistical Computing, Vienna, Austria). Data preprocessing with "dplyr" and “mice” packages, data analysis with "survey" package, association rules analysis analysis with "rules" package, visual association rules analysis with "arulesViz" package, graphing with "ggplot2" package, and so on. Joinpoint Software (Joinpoint Regression Program version 4.8.0.1, National Cancer Institute, USA) was used to study long-term trends in ASCVD prevalence, and statistical significance was defined as a two-sided *P*-value ≤ 0.05. Results from secondary endpoint analyses should be interpreted as exploratory.

## Results

### Participant characteristics

Excluding pregnant participants (*n* = 1,545) and those with missing data (*n* = 13) on ASCVD, education, insurance, and smoking status (*n* = 451), 53,083 individuals were included in the analysis, representing 212,719,813 adult Americans. Among 5729 ASCVD patients, the majority were male, elderly, White, and obesity and had a high school education or higher, middle incomes, a history of smoking, and health insurance and had arthritis, hypertension, and dyslipidemia. The participant characteristics are detailed in Table 1 and the flow chart of statistical analysis is shown in eFigure 1 (in Additional file 1). In addition, sensitivity analyses of baseline information were performed on both the full case study and the multiply interpolated dataset, and the results were generally consistent(eTable 1). The response rate declined across cycles from 76% in 1999–2000 to 48.8% in 2017–2018.

**Table 1:** Characteristics of adults with multimorbidity in ASCVD from 1999–2000 to 2017–2018

Variable	Overall	1999–2000	2001–2002	2003–2004	2005–2006	2007–2008	2009–2010	2011–2012	2013–2014	2015–2016	2017–2018	*P* value
Arthritis												0.11
No	2544(44.02)	229(46.8)	286(48.84)	268(45.2)	208(41.81)	279(43.11)	285(45.76)	255(50.24)	239(43.67)	250(39.94)	245(38.57)	
Yes	3185(55.66)	265(53.2)	280(51.16)	346(54.8)	297(58.19)	380(56.89)	353(54.24)	278(49.76)	292(56.33)	312(60.06)	382(61.43)	
Cancer												0.04
No	4544(78.45)	417(84.29)	434(78.6)	493(79.71)	404(80.82)	520(78.41)	489(76.44)	439(83.66)	422(77.77)	438(73.64)	488(74.51)	
Yes	1185(21.4)	77(15.71)	132(21.4)	121(20.29)	101(19.18)	139(21.59)	149(23.56)	94(16.34)	109(22.23)	124(26.36)	139(25.49)	
CKD												0.03
No	3375(53.33)	317(64.32)	347(60.83)	348(57.74)	271(54.4)	397(61.6)	391(63.83)	304(54.93)	296(57.13)	328(61.7)	376(62.68)	
Yes	2354(35.74)	177(35.68)	219(39.17)	266(42.26)	234(45.6)	262(38.4)	247(36.17)	229(45.07)	235(42.87)	234(38.3)	251(37.32)	
DM												< 0.001
No	3514(65.3)	330(73.45)	412(77.32)	419(70.57)	319(67.06)	379(61.75)	383(64.64)	309(63.12)	298(61.38)	321(61.78)	344(56.82)	
Yes	2215(34.7)	164(26.55)	154(22.68)	195(29.43)	186(32.94)	280(38.25)	255(35.36)	224(36.88)	233(38.62)	241(38.22)	283(43.18)	
Hyperlipidemia												0.07
No	1194(12.48)	140(14.75)	170(14.49)	142(16.15)	113(17.74)	124(14.57)	100(12.77)	101(10.37)	80(10.74)	100(11.27)	124(10.86)	
Yes	4535(81.96)	354(85.25)	396(85.51)	472(83.85)	392(82.26)	535(85.43)	538(87.23)	432(89.63)	451(89.26)	462(88.73)	503(89.14)	
Hypertension												0.35
No	1283(25.69)	128(33.71)	153(27.92)	142(24.82)	117(26.1)	142(24.33)	145(26.86)	97(24.53)	115(25.39)	121(23.6)	123(22.36)	
Yes	4446(74.31)	366(66.29)	413(72.08)	472(75.18)	388(73.9)	517(75.67)	493(73.14)	436(75.47)	416(74.61)	441(76.4)	504(77.64)	
MetS												0.34
No	4791(82.83)	420(83.35)	472(83.01)	526(83.19)	438(87.47)	530(80.49)	531(83.11)	444(80.9)	443(83.75)	455(79.38)	532(84.03)	
Yes	938(17.15)	74(16.65)	94(16.99)	88(16.81)	67(12.53)	129(19.51)	107(16.89)	89(19.1)	88(16.25)	107(20.62)	95(15.97)	
CHF												0.59
No	4447(78.79)	384(80.86)	437(78.43)	483(80.34)	379(77.49)	520(80.81)	520(83.24)	409(78.04)	410(77.92)	418(78.12)	487(81.33)	
Yes	1282(20.11)	110(19.14)	129(21.57)	131(19.66)	126(22.51)	139(19.19)	118(16.76)	124(21.96)	121(22.08)	144(21.88)	140(18.67)	
COPD												0.04
No	5058(87.46)	450(89.98)	525(92.7)	556(89.49)	451(88.65)	549(83.6)	545(86.36)	466(85.73)	469(86.65)	491(84.79)	556(87.76)	
Yes	671(12.54)	44(10.02)	41(7.3)	58(10.51)	54(11.35)	110(16.4)	93(13.64)	67(14.27)	62(13.35)	71(15.21)	71(12.24)	
Asthma												0.1
No	4635(79.8)	402(80.33)	471(82.14)	523(81.68)	412(81.05)	542(81.22)	526(81.23)	427(82.87)	404(74.3)	435(75.55)	493(78.61)	
Yes	1094(20.2)	92(19.67)	95(17.86)	91(18.32)	93(18.95)	117(18.78)	112(18.77)	106(17.13)	127(25.7)	127(24.45)	134(21.39)	
CLD												< 0.001
No	4221(65.85)	398(77.97)	464(77.53)	477(75.62)	394(74.57)	489(72.67)	480(75.77)	424(70.15)	409(76)	431(73.41)	255(35.29)	
Yes	1508(28.28)	96(22.03)	102(22.47)	137(24.38)	111(25.43)	170(27.33)	158(24.23)	109(29.85)	122(24)	131(26.59)	372(64.71)	
Obesity												0.01
No	3567(50.46)	344(61.09)	410(59.21)	409(61.75)	317(56.67)	406(58.86)	353(51.11)	322(54.87)	330(56.38)	312(49.2)	364(49.82)	
Yes	2162(40.44)	150(38.91)	156(40.79)	205(38.25)	188(43.33)	253(41.14)	285(48.89)	211(45.13)	201(43.62)	250(50.8)	263(50.18)	

Data are expressed as unweighted sizes and weighted percentages

Chi-square test is used for all comparisons

*ASCVD* atherosclerotic cardiovascular disease, *CKD* chronic kidney disease, *DM* diabetes mellitus, *CLD* Chronic liver disease, *CHF* congestive heart failure, *MetS* metabolic syndrome, *COPD* chronic obstructive pulmonary disease

### Primary outcome

#### The overall age-standardized prevalence of ASCVD stratified by multimorbidity

The overall age-standardized prevalence (ASP) of ASCVD was 7.71%(SE,0.15%). The ASP of ASCVD among individuals with any one of the 12 chronic conditions was significantly higher than those without the respective disease (*p* < 0.001 for all subgroups). The ASP of ASCVD among individuals without any chronic conditions was lower than 7.71%, ranked from low to high as follows: lipid abnormalities (5%), hypertension (5.13%), arthritis (6.23%), CKD (6.38%), CHF (6.51%), DM (6.54%), obesity (6.67%), asthma (7.13%), MetS (7.3%), COPD (7.32%), CLD (7.38%), and cancer (7.58%). However, the ASP of ASCVD in populations with any one of the 12 diseases was higher than 7.71%, ranked from high to low as follows: CHF (56.39%), COPD (17.08%), DM (13.52%), arthritis (11.78%), MetS (11.68%), asthma (11.67%), CKD (11.65%), hypertension (10.82%), cancer (10.33%), obesity (9.24%), CLD (8.73%), and dyslipidemia (8.51%). See Table 2 and eFigures 2 and 3 in Additional file 1.

**Table 2:** Trends in the prevalence of ASCVD stratified by multimorbidity from 1999–2000 to 2017–2018

Characteristic	Overall	*P* value#	1999–2000	2001–2002	2003–2004	2005–2006	2007–2008	2009–2010	2011–2012	2013–2014	2015–2016	2017–2018	*P* for trend*
Arthritis		< 0.001											
No	6.23(5.94–6.52)		6.38(5.22–7.54)	6.64(5.54–7.74)	7.01(5.78–8.24)	6.13(5.48–6.78)	6.45(5.92–6.98)	6.1(5.18–7.02)	6.52(5.52–7.52)	6(5.31–6.69)	5.42(4.56–6.28)	5.92(4.88–6.96)	0.031
Yes	11.78(10.96–12.6)		11.14(9.59–12.69)	12.95(11.03–14.87)	12.18(9.24–15.12)	12.59(9.49–15.69)	11.61(8.91–14.31)	12.18(9.42–14.94)	9.27(8.29–10.25)	10.4(7.66–13.14)	12(9.8–14.2)	13.05(10.27–15.83)	0.447
Cancer		< 0.001											
No	7.58(7.29–7.87)		7.77(6.63–8.91)	7.66(6.6–8.72)	8.53(7.24–9.82)	7.8(6.98–8.62)	7.62(6.68–8.56)	7.09(6.25–7.93)	7.87(7.05–8.69)	7.36(6.54–8.18)	6.85(6.11–7.59)	7.48(6.6–8.36)	0.081
Yes	10.33(8.92–11.74)		11.01(7.48–14.54)	11.27(7.33–15.21)	12.83(7.95–17.71)	9.14(7.06–11.22)	9.33(6.21–12.45)	11.93(6.38–17.48)	6.11(3.97–8.25)	10.49(4.2–16.78)	9.11(5.72–12.5)	12.69(8.32–17.06)	0.653
CKD		< 0.001											
No	6.38(6.07–6.69)		6.81(5.67–7.95)	6.52(5.48–7.56)	7.15(5.66–8.64)	5.89(5.18–6.6)	6.77(5.57–7.97)	6.53(5.63–7.43)	5.64(4.99–6.29)	5.95(5.09–6.81)	5.99(5.19–6.79)	6.76(5.78–7.74)	0.377
Yes	11.65(10.89–12.41)		11.41(8.9–13.92)	9.89(8.71–11.07)	14.12(10.93–17.31)	13.64(10.19–17.09)	11.06(8.85–13.27)	10.33(7.88–12.78)	13.9(11.78–16.02)	11.2(9.4–13)	10.39(8.65–12.13)	10.74(8.72–12.76)	0.784
DM		< 0.001											
No	6.54(6.27–6.81)		6.85(5.93–7.77)	7.37(6.57–8.17)	7.68(6.54–8.82)	6.82(6.06–7.58)	6.53(5.69–7.37)	6.34(5.4–7.28)	6.22(5.3–7.14)	6.01(5.25–6.77)	5.92(4.92–6.92)	6.13(5.39–6.87)	0.001
Yes	13.52(12.52–14.52)		16.61(11.63–21.59)	14.19(7.76–20.62)	14.88(10.72–19.04)	13.55(11.12–15.98)	13.97(11.27–16.67)	12.17(9.82–14.52)	13.01(10.54–15.48)	12.25(9.8–14.7)	12.34(10.18–14.5)	14.47(11.61–17.33)	0.425
Dyslipidemia		< 0.001											
No	5(4.53–5.47)		5.83(4.05–7.61)	5.18(3.69–6.67)	7.04(5.65–8.43)	6.3(4.42–8.18)	5.66(4.19–7.13)	5.09(3.21–6.97)	4.01(2.52–5.5)	3.68(2.72–4.64)	4.25(3.17–5.33)	4.11(2.78–5.44)	0.006
Yes	8.51(8.14–8.88)		8.36(7.13–9.59)	8.57(7.45–9.69)	9.46(7.72–11.2)	8.32(7.65–8.99)	8.37(7.19–9.55)	8.27(7.29–9.25)	8.6(7.82–9.38)	8.37(7.08–9.66)	8.2(7.12–9.28)	8.85(7.67–10.03)	0.966
Hypertension		< 0.001											
No	5.13(4.74–5.52)		5.94(4.35–7.53)	5.74(4.74–6.74)	5.82(4.51–7.13)	5.22(4.2–6.24)	5.07(3.99–6.15)	5.2(4.28–6.12)	4.45(3.04–5.86)	5(4.06–5.94)	4.33(2.88–5.78)	5(4.02–5.98)	0.004
Yes	10.82(10.33–11.31)		9.58(8.03–11.13)	11.72(10.01–13.43)	12.17(9.66–14.68)	10.47(9.18–11.76)	11.26(9.65–12.87)	10.76(9.41–12.11)	10.36(9.11–11.61)	9.75(8.48–11.02)	10.03(8.87–11.19)	11.98(10.67–13.29)	0.914
MetS		< 0.001											
No	7.3(7.03–7.57)		7.41(6.49–8.33)	7.51(6.49–8.53)	8.14(6.91–9.37)	7.74(6.9–8.58)	7.49(6.67–8.31)	7.12(6.36–7.88)	6.92(6.06–7.78)	7.06(6.22–7.9)	6.53(5.96–7.1)	7.39(6.55–8.23)	0.039
Yes	11.68(10.7–12.66)		11.42(7.36–15.48)	12.54(9.36–15.72)	15.01(10.17–19.85)	11.19(8.5–13.88)	11.31(8.57–14.05)	10.76(9.15–12.37)	12.79(10.22–15.36)	9.66(6.64–12.68)	11.17(8.48–13.86)	11.98(8.92–15.04)	0.452
CHF		< 0.001											
No	6.51(6.26–6.76)		6.58(5.62–7.54)	6.61(5.73–7.49)	7.47(6.18–8.76)	6.6(5.97–7.23)	6.77(6.06–7.48)	6.54(5.8–7.28)	6.34(5.54–7.14)	6.06(5.3–6.82)	5.79(5.14–6.44)	6.67(6.02–7.32)	0.163
Yes	56.39(49.53–63.25)		58.56(35.65-–81.47)	46.75(42.59–50.91)	74.27(63.08–85.46)	59.2(40.29–78.11)	56.17(42.29–70.05)	59.65(35.62–83.68)	40.56(32.23–48.89)	48.29(29.94–66.64)	67.32(47.66–86.98)	58.75(43.07–74.43)	0.702
COPD		< 0.001											
No	7.32(7.03–7.61)		7.47(6.47–8.47)	7.83(6.93–8.73)	8.33(6.96–9.7)	7.54(6.87–8.21)	7.47(6.55–8.39)	7.19(6.31–8.07)	7.26(6.46–8.06)	6.79(6.01–7.57)	6.42(5.77–7.07)	7.29(6.43–8.15)	0.014
Yes	17.08(14.2–-19.96)		30.84(18.61–43.07)	22.19(2.41–41.97)	26.99(9.1–44.88)	17.7(12.11–23.29)	13.82(9.72–17.92)	11.18(6.48–15.88)	10.71(7.69–13.73)	19.79(15.03–24.55)	18.05(13.95–22.15)	56.37(51.04–61.7)	0.488
Asthma		< 0.001											
No	7.13(6.84–7.42)		7.45(6.23–8.67)	7.66(6.74–8.58)	8.09(6.6–9.58)	7.38(6.69–8.07)	7.34(6.48–8.2)	6.97(6.11–7.83)	7.24(6.38–8.1)	6.33(5.8–6.86)	6.25(5.6–6.9)	7.07(6.31–7.83)	0.013
Yes	11.67(10.83–12.51)		10.13(8.66–11.6)	9.97(7.7–12.24)	13.92(10.8–17.04)	12.28(9.5–15.06)	11.79(9.5–14.08)	11.38(8.85–13.91)	9.77(8.3–11.24)	13.27(10.37–16.17)	11.85(8.62–15.08)	12.38(9.97–14.79)	0.351
CLD		< 0.001											
No	7.38(7.07–7.69)		7.88(6.92–8.84)	7.71(6.71–8.71)	8.49(7.18–9.8)	7.7(6.92–8.48)	7.36(6.4–8.32)	7.23(6.25–8.21)	6.9(6.02–7.78)	6.93(6.03–7.83)	6.76(5.78–7.74)	7.58(6.29–8.87)	0.012
Yes	8.73(8.18–9.28)		8.06(6.12–10)	7.98(6.22–9.74)	9.94(7.67–12.21)	9(7.12–10.88)	9.61(7.47–11.75)	8.8(8–9.6)	10.24(8.3–12.18)	8.9(7.37–10.43)	8.03(6.5–9.56)	7.98(6.78–9.18)	0.561
Obesity		< 0.001											
No	6.67(6.34–7)		7.04(5.96–8.12)	6.56(5.44–7.68)	7.96(6.59–9.33)	6.73(5.89–7.57)	7.07(6.05–8.09)	6.2(5.3–7.1)	6.49(5.59–7.39)	6.57(5.65–7.49)	5.75(4.87–6.63)	6.6(5.5–7.7)	0.073
Yes	9.24(8.77–9.71)		9.02(7.2–10.84)	10.09(7.88–12.3)	10.5(8.32–12.68)	9.67(8.38–10.96)	9.12(7.08–11.16)	9.71(8.75–10.67)	9.3(8.07–10.53)	8.1(7.02–9.18)	8.7(7.5–9.9)	9.08(7.77–10.39)	0.077

Data are expressed as age-adjusted prevalence with 95% confidence interval

*ASCVD* atherosclerotic cardiovascular disease, *CKD* chronic kidney disease, *DM* diabetes mellitus, *CLD* Chronic liver disease, *CHF* congestive heart failure, *MetS* metabolic syndrome, *COPD* chronic obstructive pulmonary disease

^*^ statistical significance of trends was assessed by calculating ABPC by Joinpoint logistic regression

^#^Chi-square test is used for subgroups comparison of variables

#### Trends in the overall ASP of ASCVD stratified by multimorbidity from 1999–2000 to 2017–2018

During the period from 1999 to 2018, the overall ASP of ASCVD showed a significant declining trend, with an average decrease rate of -0.81% (*P* = 0.035); however, subgroup analysis based on different chronic conditions revealed inconsistent trends. The fastest declining ASP trend was observed in individuals without lipid abnormalities (BPC = -3.09, *p* trend = 0.006), followed by those without DM (BPC = -1.19, *p* trend = 0.001), without hypertension (BPC = -1.17, *p* = 0.004), without asthma (BPC = -1.05, *p* trend = 0.013), without COPD (BPC = -0.87, *p* trend = 0.014), without CLD (BPC = -0.86, *p* trend = 0.012), and without arthritis (BPC = -0.81, *p* trend = 0.031). There were no significant changes in the ASCVD prevalence trend among populations with any one of the 12 chronic conditions (Table 2 and eFigures 4 -6 in Additional file 1).

#### Multimorbidity distribution in the ASCVD population

In total, 65.9% of ASCVD patients had at least four of the 12 selected chronic conditions, Among them, four and five were the most common (17.9% and 17.7%, respectively). (see Figure and eTable 2 in Additional file 1). The number of chronic conditions differed significantly according to age and sex (eFigure 7 in Additional file 1). The 20–39 years age group had the highest proportion of having one chronic condition. The 40–59 years age group had the highest proportion of having three chronic conditions. Patients over 60 years had the highest proportion of having 4–5 chronic conditions at the same time, and the difference between groups was statistically significant (χ2 = 255.1, *p* < 0.001), see eTable 3 for details. There were crossover points between males and females in the distribution of the number of individuals having five chronic conditions. Before the crossover point, the distribution of the number of multiple chronic conditions in males was higher than that in females, while the opposite was true after the crossover (χ2 = 41.09, *p* < 0.001).

#### Association rule analysis of multimorbidity in ASCVD

**Fig. 1:**
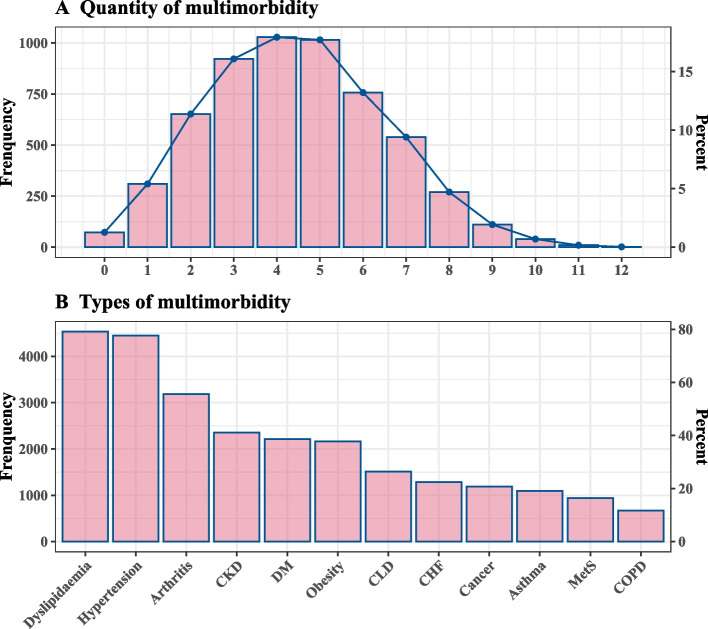
Distribution of the number and type of multimorbidity in ASCVD. ASCVD, atherosclerotic cardiovascular disease; CKD, chronic kidney disease; DM, diabetes mellitus; CLD, chronic liver disease; CHF, congestive heart failure; MetS, metabolic syndrome; COPD, chronic obstructive pulmonary disease

**Fig. 2:**
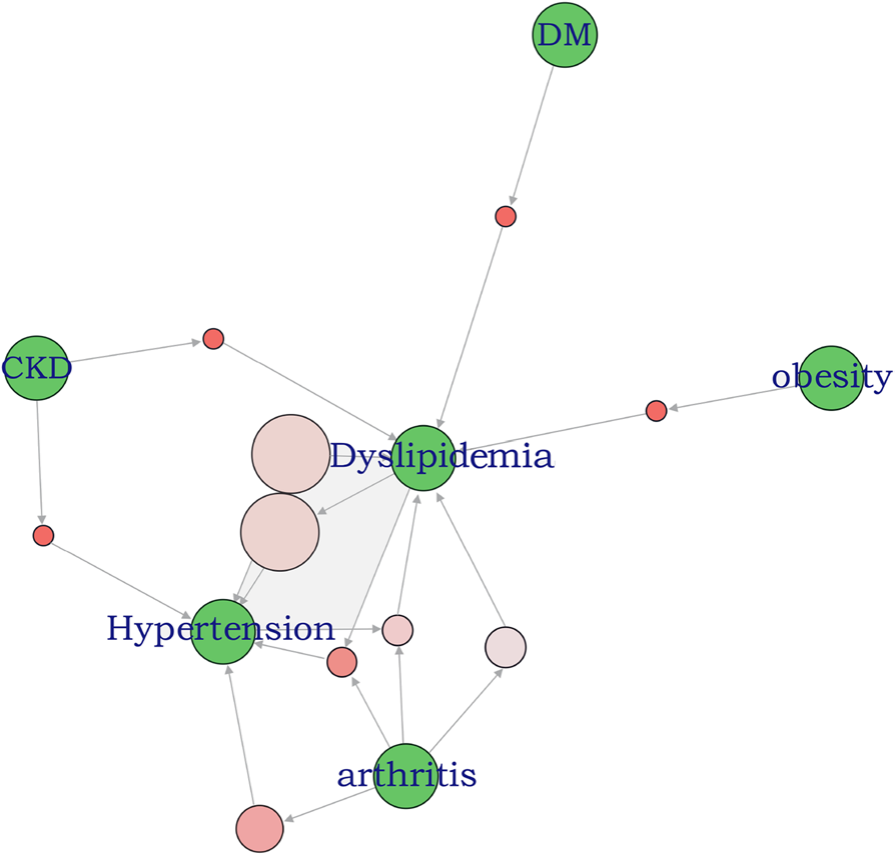
The 10 rules with the highest support show. As shown in the figure, dyslipidemia and hypertension continued to be the core diseases with strong association rules

The top five most frequent chronic conditions in the ASCVD population were dyslipidemia, hypertension, arthritis, CKD, and DM, in that order (Fig. 1). The 124 significant association rules had the highest support for a coexistence pattern between hypertension and dyslipidemia (support = 0.63); and the highest lift pertained to the coexistence of dyslipidemia, hypertension, metabolic syndrome, and DM (lift = 1.82). The top 10 rules with the highest support are shown in eTable 4 in Additional file 1 and Fig. 2, dyslipidemia and hypertension are the core diseases with strong association rules. The grouping matrix clustered the 124 rules into 10 categories, and the top left corner of the figure shows the most interesting group of rules based on lift, including metabolic syndrome, dyslipidemia, and DM (eFigure 8 in Additional file 1). The network plot also showed a strong association centered on dyslipidemia and hypertension (eFigure 9 in Additional file 1). The interactable network graph with high support (0.2), confidence (0.8), and lift (1) showed 24 association rules consisting of seven co-morbidities (dyslipidemia, hypertension, arthritis, DM, obesity, CKD, and CLD). As shown in eFigure 10 in Additional file 1, more rules included dyslipidemia, hypertension, and arthritis.

### Secondary outcomes

#### Factors associated with ASCVD

**Fig. 3:**
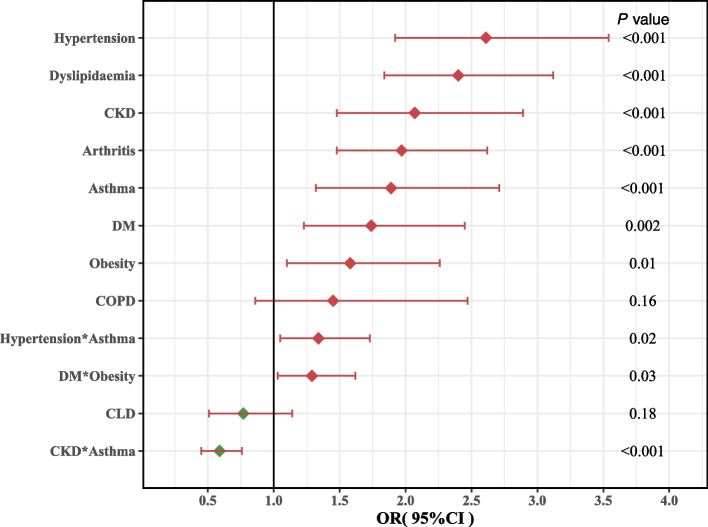
Independent association of multimorbidity with ASCVD in a multivariable logistic regression model with interaction. Model adjusted for age, sex, race/ethnicity, education, ratio of family income to poverty, insurance, and smoking status. Error bars indicate 95% CI. ASCVD, atherosclerotic cardiovascular disease; CKD, chronic kidney disease; DM, diabetes mellitus; CLD, chronic liver disease; CHF, congestive heart failure; MetS, metabolic syndrome; COPD, chronic obstructive pulmonary disease; OR, odds ratio; CI, confidence interval

The results of the univariate analysis of ASCVD correlation showed that age, sex, race, education level, poverty index, smoking, and the 12 chronic conditions were significantly correlated with ASCVD (all *p* < 0.05). In further stepwise regression multifactor analysis, insurance, MetS, and cancer were excluded, and other variables remained statistically significant (eTable 5 in Additional file 1). CHF was excluded because it was the outcome variable for most ASCVDs. Thus, 36 interaction terms for nine chronic conditions were included in the final model. The results showed that the effect values for COPD and CLD were no longer significant in the main effect term. In the other seven chronic conditions, the strength of association between the main effect term and ASCVD, including in the multifactorial model with interaction terms. was the highest for hypertension (odds ratio [OR] = 2.61; 95% confidence interval [CI], 1.92–3.54), dyslipidemia (OR = 2.4; 95% CI, 1.84–3.12), CKD (OR = 2.07; 95% CI, 1.48–2.89), arthritis (OR = 1.97; 95% CI, 1.48–2.62), asthma (OR = 1.89; 95% CI, 1.32–2.71), DM (OR = 1.74; 95% CI, 1.23–2.45), and obesity (OR = 1.58; 95% CI, 1.12–2.6), in that order. All these diseases were significantly and positively associated with ASCVD (Fig. 3 and eTable4 in Additional file 1). In addition, we added multivariate logistic regression models controlling for year fixed effect, which showed no statistically significant results compared to any other cycle, using the 1999–2000 cycle as a reference. The results of the multivariate analysis of the effect values for the various chronic diseases before and after the addition of the year effect were unchanged (eTable 6).

#### Interactions among multimorbidity in ASCVD

In the multifactor model with added interaction terms, three statistically significant multiplicative interaction terms were also observed, with positive multiplicative interactions between hypertension and asthma (OR = 1.3;4 95% CI, 1.05–1.73), and between DM and obesity (OR = 1.29; 95% CI, 1.03–1.62). Meanwhile, CKD and asthma (OR = 0.59; 95% CI, 0.45–0.76) showed a negative multiplicative interaction (eTable 7 in Additional file 1). In additive interactions, synergistic effects were observed between DM and dyslipidemia, asthma and hypertension, and asthma and arthritis, respectively, but antagonistic effects were observed between asthma and CKD (eTable 8 and eFigure 11 in Additional file 1).

## Discussion

In the current study, 66% of ASCVD patients had at least four of the 12 selected chronic conditions and 36% of patients had 4–5 chronic conditions. The prevalence of multimorbidity reported in previous studies varies widely due to different populations targeted and different diseases included. A literature review shows that the prevalence of older patients with cardiovascular disease with at least one additional morbidity ranges from 10 to 77%; the frequency of four or more additional chronic conditions ranged from 5 to 60% [26]. The overall prevalence of ASCVD was higher in populations with any one of the chronic conditions compared to that in the general population. Additionally, over the 20-year period, the decline in ASCVD prevalence was primarily observed in populations without most of the chronic conditions, while no decline was seen in the population with them. This suggests that greater attention should be given to preventing ASCVD among populations with these common chronic conditions. The top five most frequent chronic conditions in the ASCVD population were dyslipidemia, hypertension, arthritis, CKD, and DM. This is similar to the findings of previous studies. Studies have reported that the most common morbidities of cardiovascular disease in the elderly are diabetes, chronic kidney disease, anemia, chronic obstructive pulmonary disease, and dementia/cognitive impairment [26]. A cross-sectional study of Chinese patients aged 45 years and older with CVD showed hypertension (56.1%), arthritis or rheumatism (53.9%), and digestive diseases (41.5%) to be the three most common chronic disease comorbidities [14]. In a study of comorbidity patterns in adults with CVD in the United Kingdom between 2000 and 2014, the most common comorbidities were hypertension (28.9%), depression (23.0%), arthritis (20.9%), asthma (17.7%) and anxiety (15.0%). Cardiometabolic diseases and arthritis were highly prevalent in patients over 40 years of age, and psychiatric disorders were highly prevalent in patients aged 30–59 years [27]. Among the 124 association rules, there were strong associations between dyslipidemia, hypertension, MeS, and DM. In the multivariable analysis, hypertension, dyslipidemia, CKD, arthritis, asthma, DM, and obesity were significantly and positively associated with ASCVD. Additionally, interactions were observed among some chronic conditions. Chronic conditions that coexist with ASCVD may be risk factors or consequences and may share some of its pathophysiologic mechanisms or simply coexist with increasing prevalence with age [10, 28]. Among the top five most common chronic conditions in ASCVD, dyslipidemia, hypertension, and DM are all risk factors for ASCVD. This is consistent with previous research. In a 2018 analysis, 81.3%, 69.1%, and 41.7% of Medicare beneficiaries with ischemic heart disease had hypertension, dyslipidemia, and DM, respectively [11]. This confirms the importance of risk factor control for ASCVD treatment. Regarding the relationship between arthritis, CKD, and ASCVD, we found some interesting results in our multivariable analysis. In addition to well-known risk factors such as hypertension, dyslipidemia, DM, and obesity, we also found that CKD, asthma, and arthritis were significantly and positively associated with ASCVD. In addition, both multiplicative and additive interactions showed that hypertension and asthma were synergistic, while CKD and asthma were antagonistic. ASCVD and asthma share a common immune mechanistic basis [29, 30]. In a Korean study, asthma was associated with ischemic heart disease in individuals aged > 53 years [31]. Collectively, these findings support that the combination of hypertension and asthma will further increase ASCVD risk. In patients who have both hypertension and asthma, it may be necessary to pay attention or screen for the presence of ASCVD to facilitate early detection and treatment. Patients with CKD have a higher risk of developing cardiovascular disease. Early detection, staging, and treatment of CKD have a central role in initialing cardiorenal preventative therapy, and patients at a high risk of ASCVD can benefit from improvement in this regard [32]. Multiple studies have shown that asthma increases the risk of CKD, independent of obesity, DM, hypertension, and other recognized risk factors. However, treatment of asthma with steroids or nonsteroidal medications may reduce this risk. Interestingly, our study suggested that patients with both asthma and CKD had a reduced risk of ASCVD. Therefore, the relationship between these three conditions deserves further research. In addition, hypertension, DM, obesity, and dyslipidemia are risk factors for ASCVD, and co-occurrence of any two of these diseases increases the risk of ASCVD, which is consistent with the results of our interaction analysis.

Moreover, asthma and arthritis showed a synergistic additive interaction. Asthma was positively associated with rheumatoid arthritis and was a risk factor for it. Chronic mucosal airway inflammation in asthma may contribute to anti-citrullinated protein antibody development and the pathogenesis of rheumatoid arthritis. Compared with the general population, patients with rheumatoid arthritis have twice the risk of ASCVD, stroke, heart failure, and atrial fibrillation. Chronic inflammation is a common mechanism for all three diseases. Therefore, focusing on the multimorbidity of asthma, arthritis, and ASCVD, and even extending to other chronic inflammatory diseases may aid in discovering synergistic treatment options for multiple diseases.

In summary, each patient is a complex whole; the sum of multiple disease effects. Therefore, the prevention and treatment of ASCVD must also follow a comprehensive analysis of multiple disease characteristics, prognosis, drug interactions and contraindications, and social determinants of health. For some hospitalized patients or patients in Intensive Care Unit (ICU) or Emergency Room (ER), multimorbidity may receive more attention and Multi-Disciplinary Treatment (MDT) will be performed to develop a comprehensive plan. However, for more patients, especially those with multimorbidity of common chronic diseases, on the one hand, they do not know the relationship between the diseases themselves, and on the other hand, they often need to go to various departments successively when seeking medical consultation, which may make it difficult for them to get a comprehensive plan in the end. However, the management of multimorbidity is more and more important in daily and lifelong management. For example, if a patient with coronary atherosclerotic heart disease who needs to take antiplatelet medicine also suffers from arthritis and often takes NSAIDs for pain relief, long-term attention to the gastrointestinal risks associated with the combination of the two types of medications and proper management may be able to avoid a gastrointestinal hemorrhage emergency. Another example is the long-term management of patients with cardiorenal syndrome, which, if both cardiac and renal aspects are taken into account, may be able to reduce the frequency of re-hospitalization and improve the prognosis. However, how to do a good job of daily multimorbidity management is a great challenge for clinicians. Due to the current refinement and deepening of medical specialties, doctors tend to focus more on diseases in their own specialty areas and less on other areas. General practitioners, on the other hand, have a relatively less in-depth grasp of each disease. But if every patient visit is discussed jointly by multidisciplinary doctors, can the increased workload and healthcare costs be affordable? Is it possible to continue to train General practitioners in depth and focus on the management of multimorbidity? Or can we explore better forms of multidisciplinary discussion? This may requires increased awareness at the public health level, in-depth and extensive research, and increased investment. Thus, it can effectively improve the cognition of both doctors and patients on multimorbidity, complete the comprehensive diagnosis and treatment of multimorbidity, optimize the management of patients with complex conditions, and cover the whole course of multimorbidity prevention and treatment. However, in any case, the collaborative management of ASCVD and its multimorbidity is crucial.

This study had some limitations. First, self-reporting of some chronic conditions and potential misclassification of drug use meant potential misclassification bias could not be ruled out. Second, partially missing data on chronic conditions such as CKD and DM may have reduced the estimates of disease prevalence. Third, some confounding and mediating factors may not have been considered. Finally, other chronic conditions and social determinants of health could not be included in this study owing to limited NHANES survey content.

## Conclusion

The overall prevalence of ASCVD significantly decreased over the 20-year survey period. However, this reduction was primarily observed in populations without dyslipidemia, DM, hypertension, asthma, COPD, and arthritis. The majority of ASCVD patients had four or more concurrent chronic conditions. Dyslipidemia and hypertension were the core diseases among chronic conditions with strong association rules. In addition to traditional risk factors such as hypertension, dyslipidemia, DM, and obesity, attention should also be paid to the multimorbidity of asthma, arthritis, CKD, and ASCVD.

### Supplementary Information

**Additional file 1:** **Appendix 1.** Diagnostic criteria for 12 chronic diseases. **Appendix 2. **eTables and eFigures.

## Data Availability

The datasets underlying this article were derived from sources in the public domain: https://wwwn.cdc.gov/nchs/nhanes/continuousnhanes/default.aspx?Cycle=1999–2018.

## Abbreviations

ASCVD: Atherosclerotic cardiovascular disease
NHANES: National Health and Nutrition Examination Survey
DM: Diabetes mellitus
COPD: Chronic obstructive pulmonary disease
ACC: American College of Cardiology
CLD: Chronic liver disease
CKD: Chronic kidney disease
MetS: Metabolic syndrome
CHF: Congestive heart failure
AIC: Akaike information criterion
ASP: Age-standardized prevalence
RERI: Relative excess risk due to interaction
AP: Attributable proportion due to interaction
S: Synergy index
