# Supplementary material for: Epidemiology of multimorbidity associated with atherosclerotic cardiovascular disease in the United States, 1999–2018
Source: BMC Public Health. 2024 Jan 23;24:267. doi: 10.1186/s12889-023-17619-y (PMC10804461; doi:10.1186/s12889-023-17619-y)
Supplement: Supplementary file 1 — Additional file 1: Appendix 1. Diagnostic criteria for 12 chronic diseases. Appendix 2. eTables and eFigures. [file 12889_2023_17619_MOESM1_ESM.docx]

**Supplementary Appendix**

**Appendix 1. Diagnostic criteria for 12 chronic diseases**

**Appendix 2. eTables and eFigures**

**eFigure 1. Flow chart**

**eTable 1：Baseline comparison of full case and imputed data**

**eFigure 2. Distribution of the overall ASP of ASCVD stratified by multimorbidity (shown in forest map)**

**eFigure 3. Distribution of the overall prevalence of ASCVD stratified by multimorbidity (shown in bar chart)**

**eFigure 4. Trends in the prevalence of ASCVD and stratified by dyslipidemia and hypertension from 1999-2000 to 2017-2018**

**eFigure 5. Trends in the prevalence of ASCVD stratified by DM and COPD from 1999-2000 to 2017-2018**

**eFigure 6. Trends in the prevalence of ASCVD stratified by arthtics,CLD and asthma from 1999-2000 to 2017-2018**

**eTable2. Quantity distribution of multimorbidity in ASCVD**

**eTable 3 Quantity distribution of multimorbidity in ASCVD stratified by age group**

**eFigure7. Quantity distribution of multimorbidity in ASCVD stratified by age and sex**

**eTable4. The top 10 rules with the highest support**

**eFigure 8. Interactive mode for grouped matrix visualization for 124 rules**

**eFigure9. Graph-based visualization with multimorbidity and 124 rules as vertices.**

**eFigure10. Interactable network diagram with 24 rules**

**eTable 5. Factors associated with ASCVD in logistic regression model**

**eTable 6：Multivariate logistic regression with year fixed effect included**

**eTable7. Multiplicative interactions among multimorbidity in ASCVD in a multivariable logistic regression model**

**eTable 8. Additive interactions among multimorbidity in ASCVD in a multivariable logistic regression model**

**eFigure 11. Additive interactions between multimorbidity in ASCVD (shown in bar chart)**

**Appendix 1. Diagnostic criteria for 12 chronic diseases**

1. **Chronic liver disease, CLD**

CLD was defined as having any one of the following:

a: AALD was defined using clinical laboratory data and self-reported alcohol use; Among adults ($20 years of age) with alcohol use (28 g/d in women and 42 g/d in men in the past 12 months) and elevated liver enzymes (alanine aminotransferase (ALT) >40 U/L or aspartate aminotransferase (AST) >37 U/L in men and ALT or AST >31 U/L in women. ^1^

b: NAFLD: NAFLD was defined using the Fatty Liver Index (FLI) score ≥60.^2^

c: MAFLD: MAFLD was defined by controlled attenuation parameter (CAP) scores of >=248 dB/m in absence of excessive alcohol use and viral hepatitis.^3^

1. **Arthritis**

The diagnosis of arthritis was based on self-reports from patients in questionnaires and including osteoarthritis, degenerative arthritis, rheumatoid arthritis or psoriatic arthritis.

1. **Chronic kidney disease CKD^4^**

With reference to KDIGO 2021 Guideline：CKD was diagnosed by:

a. Decreased renal function：GFR<60ml/min/1.73m2(stage of GFR: G3a-G5);

b. Renal damage: urinary albumin creatinine ratio（ACR）＞30mg/g.

1. **Asthma**

Asthma was defined as having any one of the following:

a.Asthma was diagnosed by : ever been told have asthma or had asthma attack in past year;

b.Use anti-asthmatic medications;

c. Use drug: selective anyone of phosphodiesterase-4 inhibitors, mast cell stabilizers, leukotriene modifiers, inhaled corticosteroids and age < 40years old, no smoking history, no chronic bronchitis or emphysema.

1. **Chronic Obstructive Pulmonary Disease，COPD^5^**

Hyperlipidemia was defined as having any one of the following:

a. COPD was diagnosed by: FEV1/FVC < 0.7 Post-Bronchodilator;

b. Ever been told have COPD;

c. Use medications: selective anyone of phosphodiesterase-4 inhibitors, mast cell stabilizers, leukotriene modifiers, inhaled corticosteroids and age above 40 years, with smoking history or chronic bronchitis.

1. **Diabetes Mellitus，DM^6^**

DM was defined as having any one of the following:

a. Ever been told have diabetes;

b. Glycohemoglobin HbA1c (%) > 6.5;

c. Fasting glucose (mmol/l) >= 7.0;

d. Random blood glucose (mmol/l) >= 11.1;

e. Two-hour OGTT blood glucose (mmol/l) >= 11.1;

f. Use of diabetes medication or insulin.

1. **Dyslipidemia^7^**

Dyslipidemia was defined as having any one of the following:

a. high triglycerides：TG≥150md/dl;

b. Hypercholesterolemia: TC≥200mg/dl[5.18mmol/l], LDL≥130mg/dl[3.37mmol/l];

HDL＜40mg/dl[1.04mmol/l]（male）, 50mg/dl[1.30mmol/l](female);

c. To use lipid-lowering medications.

1. **Hypertension^8^**

Hypertension was defined as SBP level of 140mmHg or higher, DBP level of 90 mmHg or higher, or self-reported use of antihypertensive medications.

1. **Metabolic Syndrome，Mets^9-10^**

Metabolic syndrome was defined as having any three of the following: Waist circumference ＞ 102 cm for men or 88 cm for women, Raised triglycerides >150 mg/dL (1.7 mmol/L),Reduced HDL-cholesterol<40 mg/dL (1.03 mmol/L) in men or <50 mg/dL (1.29 mmol/L) in women, Systolic ≥130 mm Hg or Diastolic ≥85 mm Hg, Treatment of previously diagnosed hypertension. Raised fasting plasma glucose，Fasting plasma glucose ≥100 mg/dL (5.6 mmol/L) or take diabetes medication.

1. **Congestive heart failure，CHF**

Diagnosis of CHF: Based on self-report of patients in questionnaires.

1. **Cancer**

Diagnosis of cancer: Based on self-report of patients in questionnaires.

1. **Obesity**

Diagnosis of obesity: Based on Body Mass Index (BMI)≥30 kg/ m^2^.

**References**

1. Rattan P, Penrice DD, Ahn JC, et al. Inverse Association of Telomere Length With Liver Disease and Mortality in the US Population. Hepatol Commun. 2022 Feb;6(2):399-410.
2. Bedogni G, Bellentani S, Miglioli L, et al. The Fatty Liver Index: a simple and accurate predictor of hepatic steatosis in the general population. BMC Gastroenterol. 2006 Nov 2; 6:33.
3. Eslam M, Newsome PN, Sarin SK, et al. A new definition for metabolic dysfunction-associated fatty liver disease: An international expert consensus statement. J Hepatol. 2020 Jul;73(1):202-209.
4. Rovin, Brad H, Adler, et al. Executive summary of the KDIGO 2021 Guideline for the Management of Glomerular Diseases. Kidney International, 2021. 100(4): p. 753-779.
5. Wan ES, Balte P, Schwartz JE, et al. Association Between Preserved Ratio Impaired Spirometry and Clinical Outcomes in US Adults. JAMA. 2021 Dec 14;326(22):2287-2298.
6. American Diabetes Association. 2. Classification and Diagnosis of Diabetes: Standards of Medical Care in Diabetes-2021. Diabetes Care. 2021 Jan;44(Suppl 1): S15-S33.
7. Kammerlander AA, Mayrhofer T, Ferencik M, et.al. PROMISE Investigators. Association of Metabolic Phenotypes With Coronary Artery Disease and Cardiovascular Events in Patients With Stable Chest Pain. Diabetes Care. 2021 Apr;44(4):1038-1045.
8. Chobanian AV, Bakris GL, Black HR, et al. The seventh report of the Joint National Committee on prevention, detection, evaluation, and treatment of high blood pressure: the JNC 7 report. JAMA.2003;289(19):2560-2572.
9. Alberti KG, Zimmet P, Shaw J.IDF Epidemiology Task Force Consensus Group. The metabolic syndrome--a new worldwide definition. Lancet. 2005 Sep 24-30;366(9491):1059-62.
10. Alberti KG, Eckel RH, Grundy SM, et al; International Diabetes Federation Task Force on Epidemiology and Prevention; Hational Heart, Lung, and Blood Institute; American Heart Association; World Heart Federation; International Atherosclerosis Society; International Association for the Study of Obesity. Harmonizing the metabolic syndrome: a joint interim statement of the International Diabetes Federation Task Force on Epidemiology and Prevention; National Heart, Lung, and Blood Institute; American Heart Association; World Heart Federation; International Atherosclerosis Society; and International Association for the Study of Obesity. Circulation. 2009 Oct 20;120(16):1640-5.

**Appendix 2. eTables and eFigures**

**eFigure 1. Flow chart**


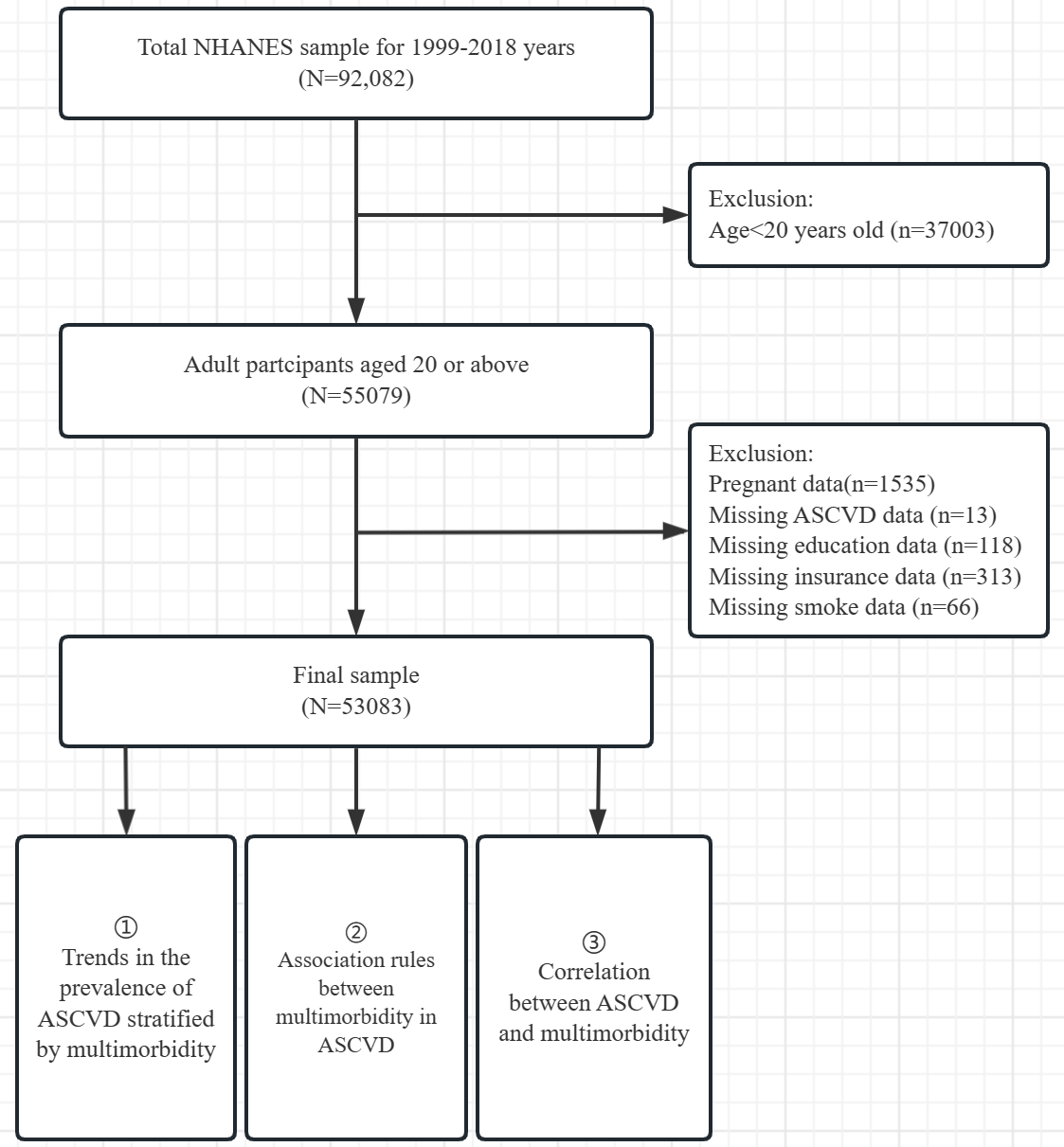


ASCVD, atherosclerotic cardiovascular disease

We defined 12 multimorbidities associated or potentially associated with ASCVD from the list of chronic conditions, including arthritis, cancer, CKD, DM, dyslipidemia, hypertension, MetS, CHF, CLD, asthma, COPD, and obesity. The other eight diseases are not considered, including: Cardiac arrhythmias, Autism spectrum disorder, Dementia (including Alzheimer’s and other senile dementias), Depression, Diabetes, Hepatitis, Human immunodeficiency virus (HIV), Osteoporosis, Schizophrenia, Substance abuse disorders (drug and alcohol). The reason why the above eight disorders were not considered is that, on the one hand, there is no diagnostic information in the NHANES database for five diseases, such as cardiac arrhythmia、autism spectrum disorder, dementia, schizophrenia and substance abuse disorders; on the other hand, from 1999-2018, there is too much missing information for three diseases including depression, Human immunodeficiency virus (HIV) and Osteoporosis .

**eTable 1：Baseline comparison of full case and imputed data**

| Character | complete cases | imputed cases |
| --- | --- | --- |
| n(N) | 45247(186298082) | 53083(212719813) |
| Age(year) | 46.90(0.19) | 47.30(0.18) |
| Pir | 3.00(0.03) | 2.99(0.03) |
| BMI(kg/m^2^) | 28.79(0.06) | 28.77(0.06) |
| Age_group |  |  |
| 20-39 | 37.02 | 36.68 |
| 40-49 | 20.35 | 19.89 |
| 50-59 | 18.43 | 18.12 |
| 60-69 | 12.85 | 12.86 |
| >=70 | 11.34 | 12.45 |
| Sex |  |  |
| Female | 51.21 | 51.28 |
| Male | 48.79 | 48.72 |
| Race/ethinicity |  |  |
| Non-Hispanic White | 69.2 | 68.22 |
| Non-Hispanic Black | 10.99 | 11.19 |
| Hispanic | 13.07 | 13.66 |
| Other Race | 6.74 | 6.94 |
| Education |  |  |
| Less than high school | 16.61 | 17.52 |
| High school diploma | 24.01 | 24.11 |
| More than high school | 59.38 | 58.37 |
| Ratio of family income to poverty | |  |
| <1.3 | 21.25 | 21.61 |
| 1.3-3.49 | 35.87 | 35.95 |
| 3.5-5 | 42.89 | 42.45 |
| Insurance |  |  |
| No | 17.38 | 17.63 |
| Yes | 82.62 | 82.37 |
| Smoke status |  |  |
| former | 24.72 | 24.65 |
| never | 53.57 | 53.61 |
| now | 21.72 | 21.74 |
| BMI_group |  |  |
| <25 | 31.15 | 31.21 |
| 25-29.99 | 35.64 | 35.45 |
| >=30 | 33.21 | 33.34 |

**eFigure 2.** **Distribution of the overall ASP of ASCVD stratified by multimorbidity** **(shown in forest map)**


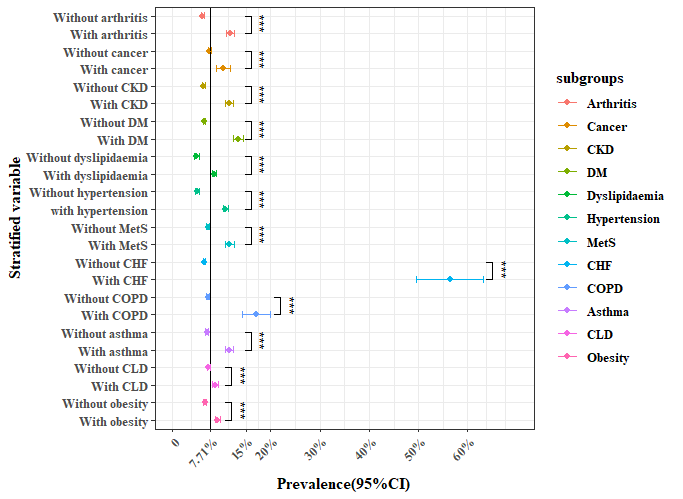


ASCVD, atherosclerotic cardiovascular disease; 95% CI, 95% confidence interval; CKD, chronic kidney disease ; DM, diabetes mellitus ; CLD, Chronic liver disease ;CHF, congestive heart failure; MetS, metabolic syndrome ;COPD, chronic obstructive pulmonary disease; error bars indicate 95% confidence intervals;

The 7.71% was the overall age-standardized prevalence of ASCVD

**eFigure 3. Distribution of the overall prevalence of ASCVD stratified by multimorbidity** **(shown in bar chart)**


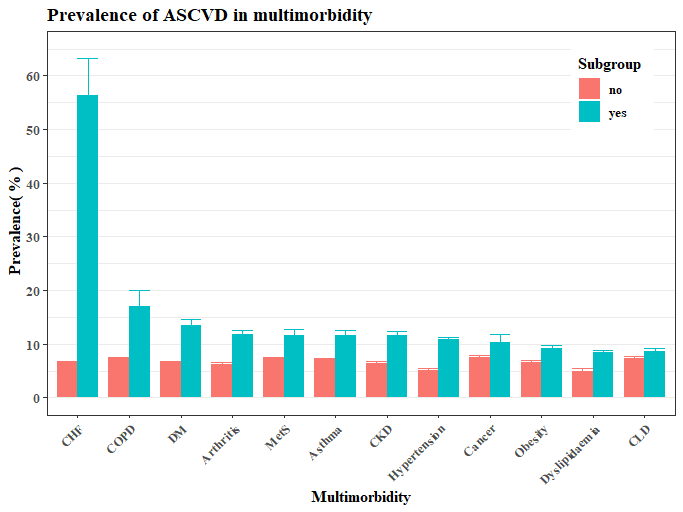


ASCVD, atherosclerotic cardiovascular disease; CKD, chronic kidney disease; DM, diabetes mellitus ; CLD, Chronic liver disease ;CHF, congestive heart failure; MetS, metabolic syndrome ;COPD, chronic obstructive pulmonary disease; error bars indicate 95% confidence intervals

**eFigure 4. Trends in the prevalence of ASCVD and stratified by dyslipidemia and hypertension from 1999-2000 to 2017-2018**


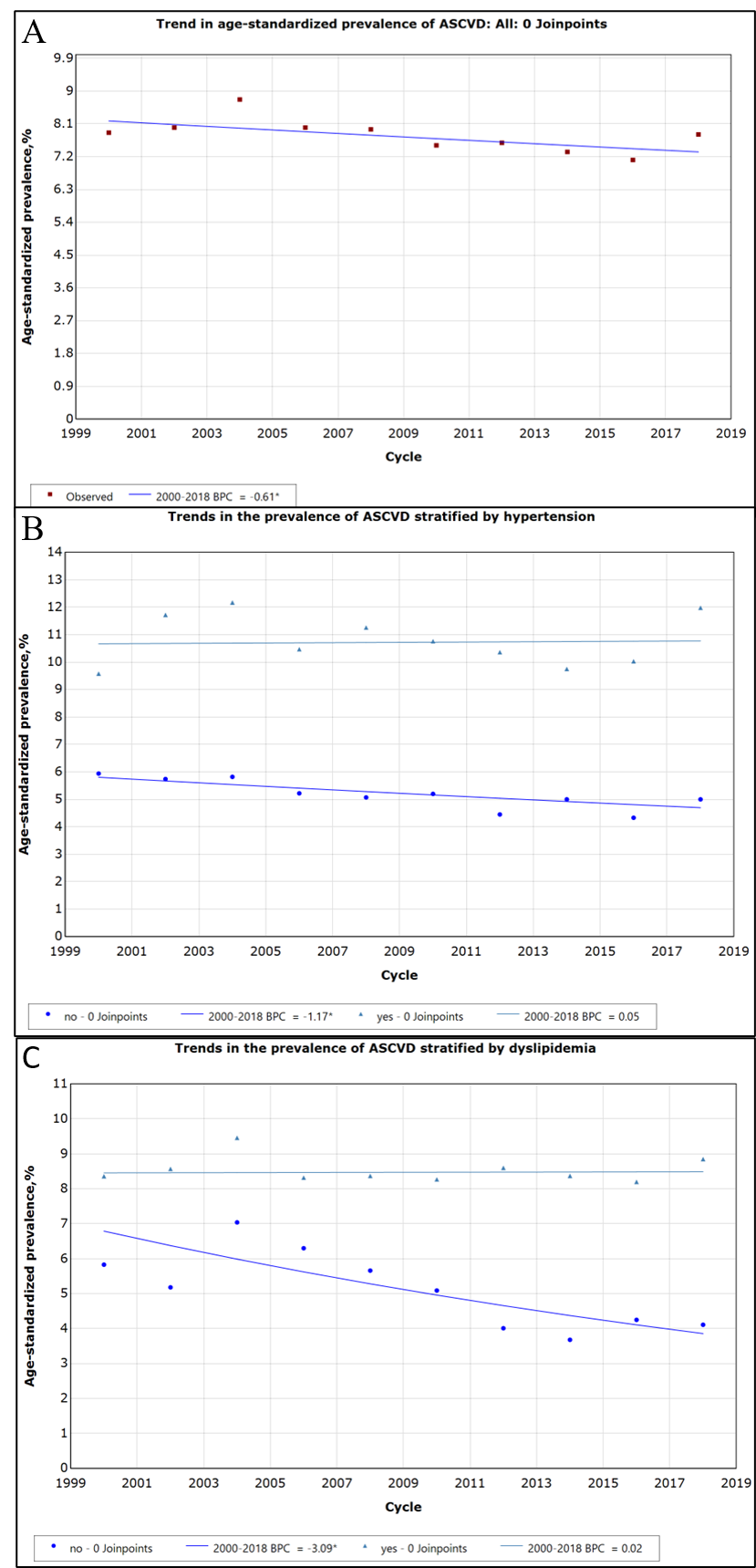


ASCVD, atherosclerotic cardiovascular disease; DM, diabetes mellitus

*Indicates that the Annual Biannual Percent(ABPC) is significantly different from zero at the α= 0.05 level.

**eFigure 5.** **Trends in the prevalence of ASCVD stratified by DM and COPD from 1999-2000 to 2017-2018**


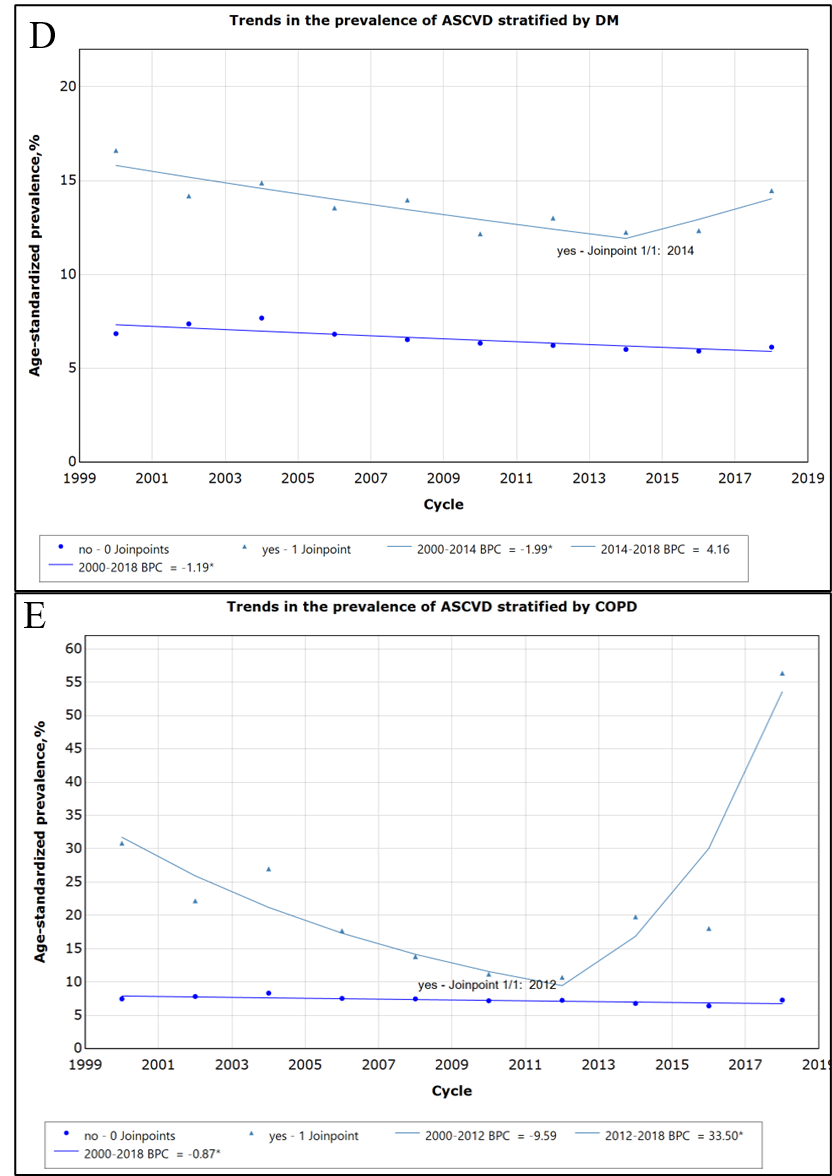


ASCVD, atherosclerotic cardiovascular disease; COPD, chronic obstructive pulmonary disease; CLD, Chronic liver disease

*Indicates that the Annual Biannual Percent(ABPC) is significantly different from zero at the α= 0.05 level.

**eFigure 6. Trends in the prevalence of ASCVD stratified by arthtics,CLD and asthma from 1999-2000 to 2017-2018**
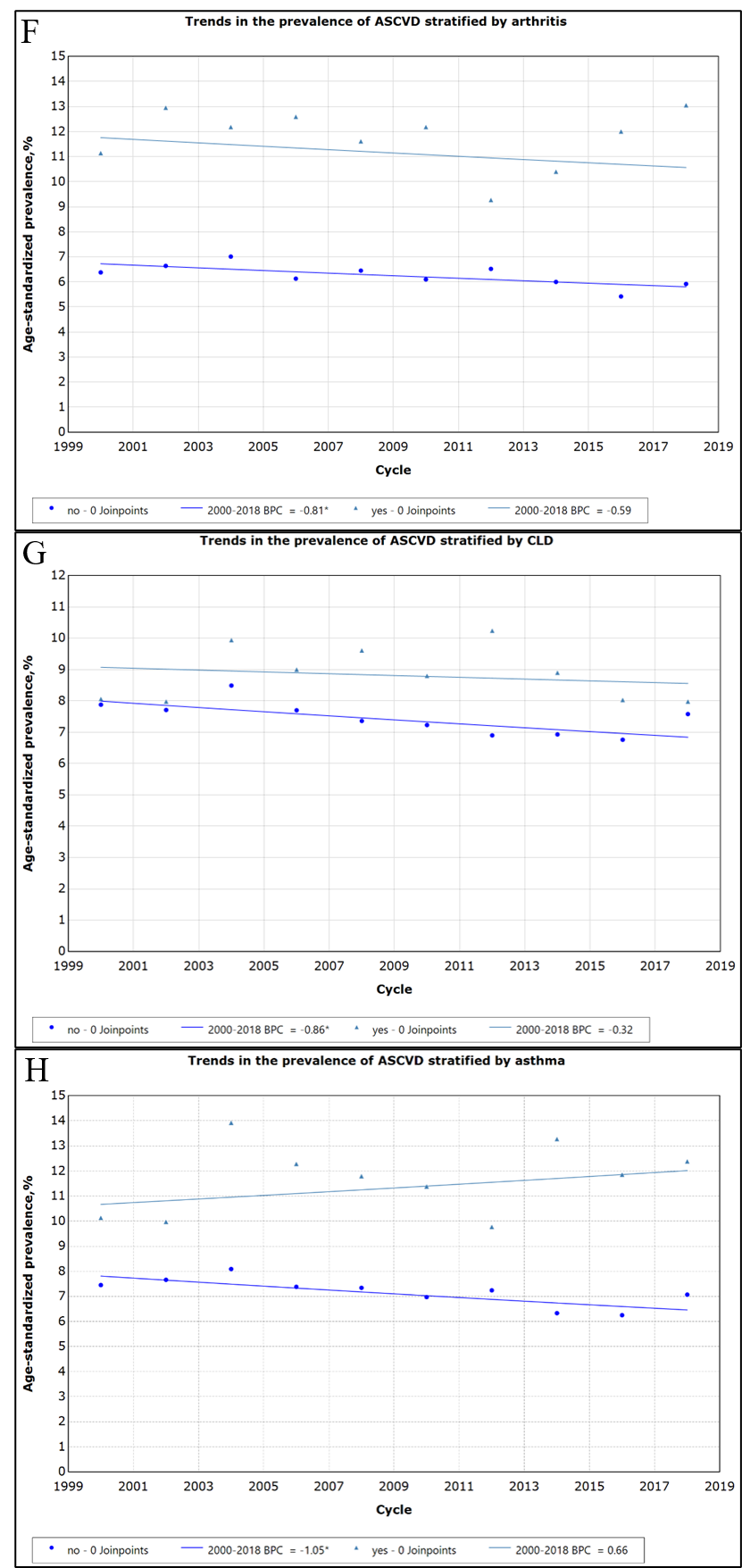


ASCVD, atherosclerotic cardiovascular disease; CLD, Chronic liver disease

*Indicates that the Annual Biannual Percent(ABPC) is significantly different from zero at the α= 0.05 level.

**eTable2. Quantity distribution of multimorbidity in ASCVD**

| Quantity | Frequency | Percent(%) | Cumulative percent (%) | Residual percent(%)^*^ |
| --- | --- | --- | --- | --- |
| 0 | 73 | 1.27 | 1.27 | 100 |
| 1 | 310 | 5.41 | 6.68 | 98.73 |
| 2 | 652 | 11.38 | 18.06 | 93.32 |
| 3 | 922 | 16.09 | 34.15 | 81.94 |
| 4 | 1028 | 17.94 | 52.09 | 65.85 |
| 5 | 1015 | 17.72 | 69.81 | 47.91 |
| 6 | 757 | 13.21 | 83.02 | 30.19 |
| 7 | 539 | 9.41 | 92.43 | 16.98 |
| 8 | 270 | 4.71 | 97.14 | 7.57 |
| 9 | 111 | 1.94 | 99.08 | 2.86 |
| 10 | 40 | 0.7 | 99.78 | 0.92 |
| 11 | 10 | 0.18 | 99.96 | 0.22 |
| 12 | 2 | 0.04 | 100 | 0.04 |

* In this column, the formula for the residual percent = 100% - the cumulative percent in the (quantity-1) row. For example, 65.85% in this column = 100% - 34.15% in the previous column. It indicates the percentage of the ASCVD population with a combination of 4 or more chronic conditions.

**eTable 3 Quantity distribution of multimorbidity in ASCVD stratified by age group**

| Frequency(n) | Age group（n %) | | | | |
| --- | --- | --- | --- | --- | --- |
|  | 20-39 | 40-49 | 50-59 | 60-69 | ≥70 |
| 0 | 4719(27.46) | 1215(13.54) | 473(5.87) | 272(3.09) | 239(2.37) |
| 1 | 5298(30.82) | 2277(25.38) | 1477(18.34) | 921(10.46) | 761(7.56) |
| 2 | 3366(19.58) | 1932(21.53) | 1649(20.47) | 1558(17.7) | 1475(14.66) |
| 3 | 2059(11.98) | 1424(15.87) | 1495(18.56) | 1659(18.85) | 2003(19.9) |
| 4 | 1037(6.03) | 1008(11.23) | 1204(14.95) | 1551(17.62) | 2013(20) |
| 5 | 437(2.54) | 593(6.61) | 843(10.47) | 1238(14.06) | 1612(16.02) |
| 6 | 194(1.13) | 307(3.42) | 503(6.24) | 807(9.17) | 1010(10.04) |
| 7 | 59(0.34) | 133(1.48) | 280(3.48) | 491(5.58) | 556(5.52) |
| 8 | 17(0.1) | 60(0.67) | 90(1.12) | 203(2.31) | 270(2.68) |
| 9 | 2(0.01) | 18(0.2) | 30(0.37) | 85(0.97) | 86(0.85) |
| 10 | — | 6(0.07) | 10(0.12) | 13(0.15) | 29(0.29) |
| 11 | — | — | 1(0.01) | 5(0.06) | 8(0.08) |
| 12 | — | — | — | — | 2(0.02) |

**eFigure7.** **Quantity distribution of multimorbidity in ASCVD stratified by age and sex**


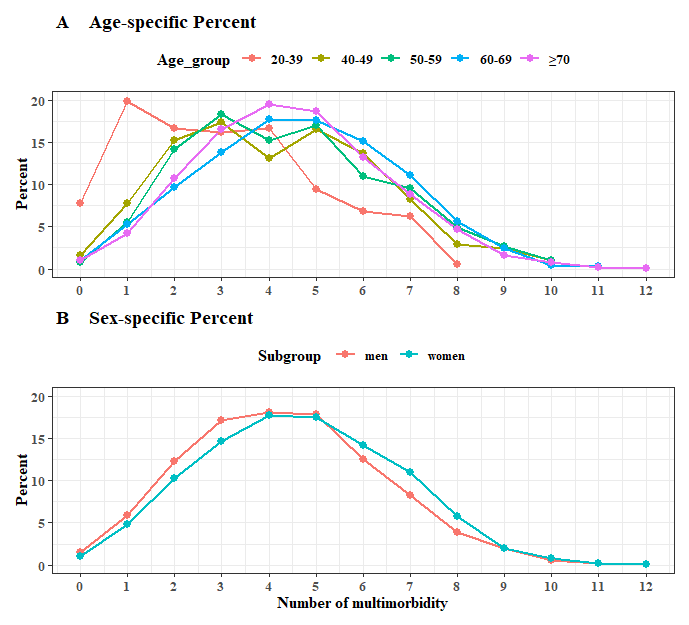


In Figure A, the age group 20-39 years had the highest proportion of 1 co-occurring chronic disease. The age groups 40-49 years and 50-59 years had the highest proportion of 3co-occurring chronic diseases. Patients aged 60-69 years and over 70 years had the highest proportion of suffering from 4-5 co-occurring chronic diseases at the same time, and the difference between groups was statistically significant (χ2=255.1, *P*<0.001). In Figure B, there were crossover points in the distribution of the number of 5 co-occurring chronic diseases between males and females. Before the crossover point, the distribution of the number of co-occurring chronic disease in males was higher than that in females, and after the crossover, the distribution of the number of co-occurring chronic disease in females was higher than that in males, with statistical significance between groups (χ2=41.09, *P*<0.001).

**eTable4. The top 10 rules with the highest support**

| **LHS** | **RHS** | **Support** | **Confidence** | **Lift** | **Count** |
| --- | --- | --- | --- | --- | --- |
| hypertension | dyslipidemia | 0.63 | 0.81 | 1.01 | 3586 |
| arthritis | hypertension | 0.46 | 0.82 | 1.04 | 2603 |
| arthritis | dyslipidemia | 0.45 | 0.8 | 1.01 | 2551 |
| arthritis, dyslipidemia | hypertension | 0.37 | 0.83 | 1.05 | 2113 |
| CKD | hypertension | 0.36 | 0.86 | 1.09 | 2013 |
| CKD | dyslipidemia | 0.36 | 0.87 | 1.09 | 2057 |
| obesity | dyslipidemia | 0.34 | 0.88 | 1.1 | 1908 |
| DM | dyslipidemia | 0.34 | 0.86 | 1.07 | 1897 |
| DM | hypertension | 0.33 | 0.84 | 1.07 | 1858 |
| obesity | hypertension | 0.32 | 0.83 | 1.06 | 1802 |

ASCVD, atherosclerotic cardiovascular disease; LHS, left-hand-side; RHS, right-hand-side; CKD: chronic kidney disease; DM: diabetes mellitus.

Set the lift to 1, without restricting the LHS and RHS of the rule.

**eFigure 8. Interactive mode for grouped matrix visualization for 124 rules**


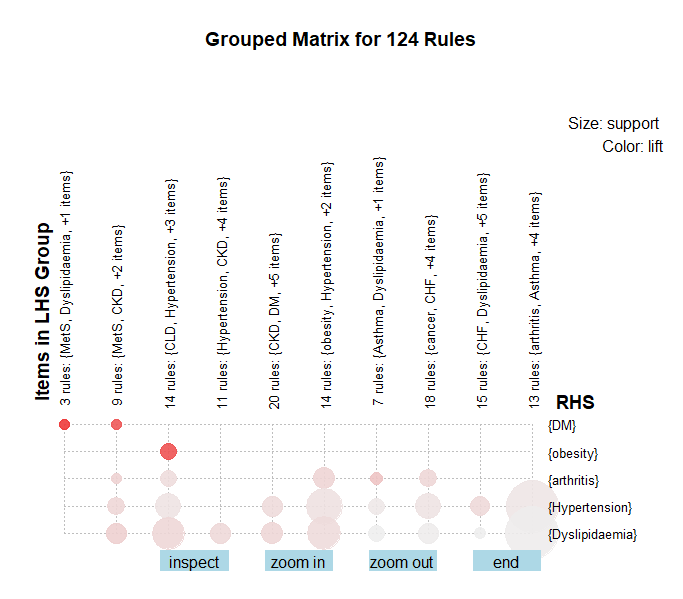


ASCVD, atherosclerotic cardiovascular disease; CKD: chronic kidney disease; DM: diabetes mellitus; CLD: Chronic liver disease; CHF: congestive heart failure; MetS: metabolic syndrome

124 rules clustered into 10 categories. The color of the balloons represented the aggregated interest measure in the group with a certain consequent and the size of the balloon shows the aggregated support. furthermore, placing the most interesting group in the top left corner. As shown in the figure, Category 1 consists of 3 rules, including MetS, Dyslipidemia and DM.

**eFigure9. Graph-based visualization with multimorbidity and 124 rules as vertices.**


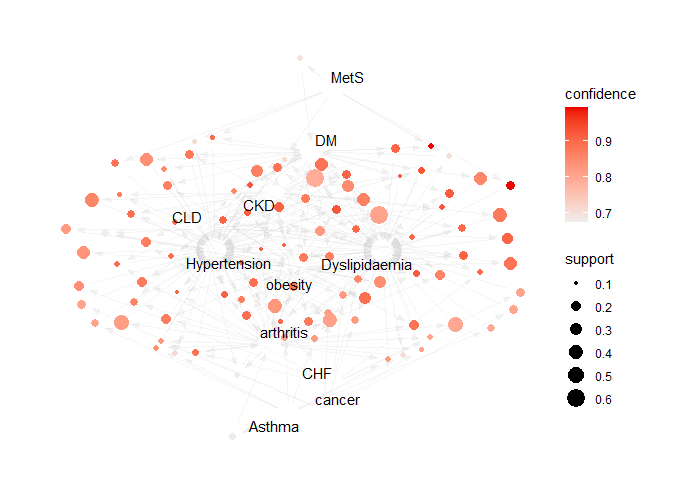


ASCVD, atherosclerotic cardiovascular disease; CKD: chronic kidney disease; DM: diabetes mellitus; CLD: Chronic liver disease; CHF: congestive heart failure; MetS: metabolic syndrome;

This representation focused on how the rules are composed of individual multimorbidity and showed which rules share multimorbidity. The network diagram demonstrated a strong association with hypertension and dyslipidemia as the core.

**eFigure10. Interactable network diagram with 24 rules**


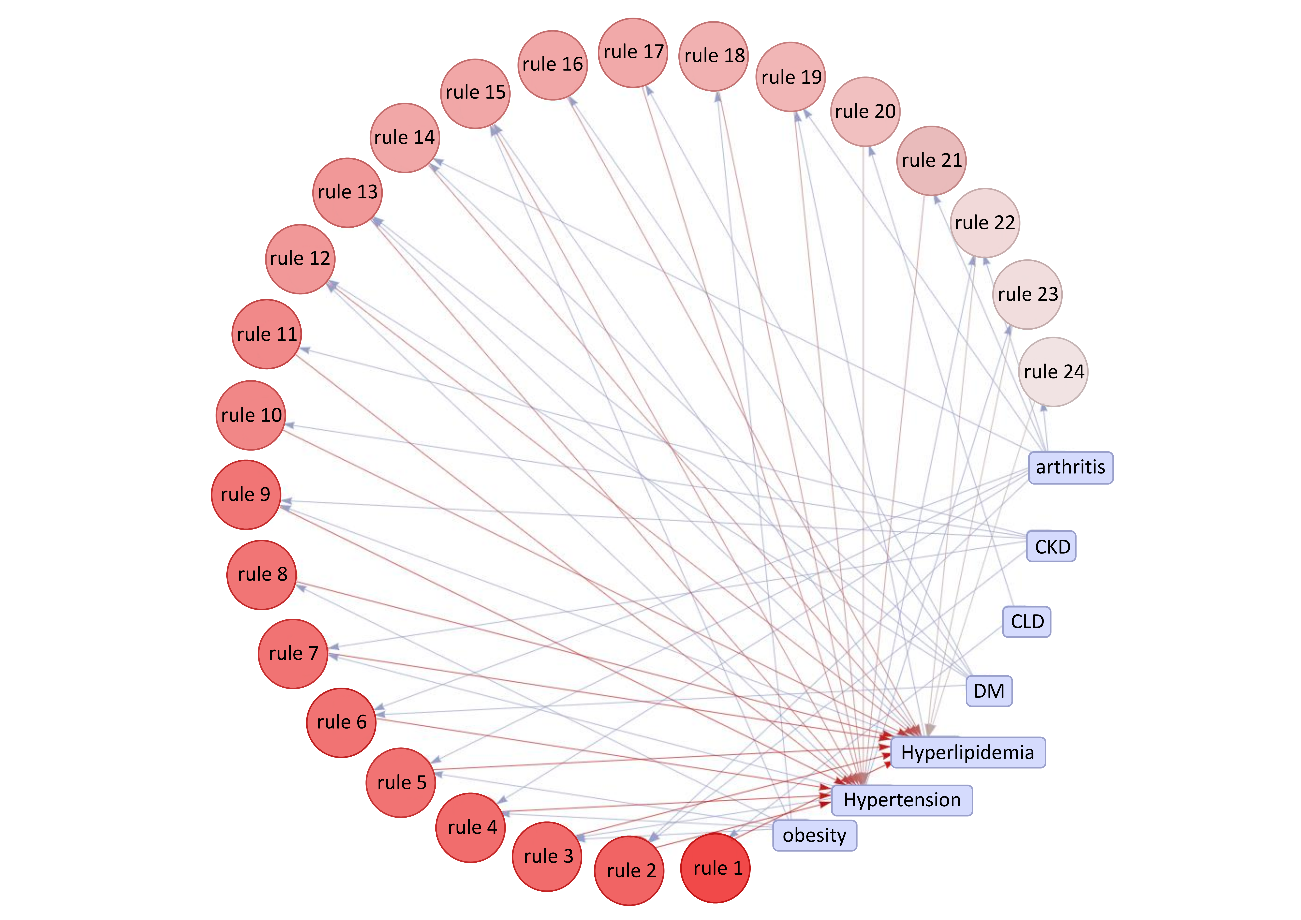


CKD: chronic kidney disease; DM: diabetes mellitus; CLD: Chronic liver disease;

A further increase in support of 0.2, confidence of 0.8, and lift of 1 yielded 24 rules consisting of 7 co-morbidities. As shown in the figure, dyslipidemia and hypertension produced the most rules.

**eTable 5.** **Factors associated with ASCVD in logistic regression model**

| **Variables** | **Univariate** | | **Multivariable** | |
| --- | --- | --- | --- | --- |
|  | **OR (95% CI)** | ***P* value** | **aOR (95% CI)** | ***P* value** |
| Age group(y) |  |  |  |  |
| 20-39 | ref | ref | ref | ref |
| 40-49 | 3.75( 3.04- 4.64) | <0.001 | 2.82(2.27- 3.51) | <0.001 |
| 50-59 | 8.33( 6.84-10.15) | <0.001 | 4.71(3.86- 5.74) | <0.001 |
| 60-69 | 18.98(15.79-22.82) | <0.001 | 8.45(6.97-10.23) | <0.001 |
| >=70 | 36.66(30.67-43.82) | <0.001 | 13.86(11.46-16.77) | <0.001 |
| Sex |  |  |  |  |
| Female | ref | ref | ref | ref |
| Male | 1.29(1.19-1.40) | <0.001 | 1.77(1.61- 1.96) | <0.001 |
| Race/ethinicity |  |  |  |  |
| Non-Hispanic White | ref | ref | ref | ref |
| Non-Hispanic Black | 0.85(0.79-0.92) | <0.001 | 0.86(0.78- 0.95) | 0.003 |
| Hispanic | 0.47(0.42-0.52) | <0.001 | 0.66(0.59- 0.76) | <0.001 |
| Other Race | 0.77(0.64-0.94) | 0.01 | 1.02(0.83- 1.24) | 0.88 |
| Education |  |  |  |  |
| Less than high school | ref | ref | ref | ref |
| High school diploma | 0.74(0.67-0.82) | <0.001 | 0.94(0.84- 1.05) | 0.27 |
| More than high school | 0.49(0.44-0.54) | <0.001 | 0.83(0.74- 0.92) | 0.001 |
| Ratio of family income to poverty |  |  |  |  |
| <1.3 | ref | ref | ref | ref |
| 1.3-3.49 | 0.84(0.77-0.91) | <0.001 | 0.71(0.64- 0.78) | <0.001 |
| 3.5-5 | 0.52(0.47-0.58) | <0.001 | 0.55(0.49- 0.62) | <0.001 |
| Insurance |  |  |  |  |
| No | ref | ref |  |  |
| Yes | 2.48(2.20- 2.81) | 0.06 | - | - |
| Smoke status |  |  |  |  |
| Never | ref | ref | ref | ref |
| Former | 2.40(2.20-2.61) | <0.001 | 1.29(1.18- 1.41) | <0.001 |
| Now | 1.42(1.28-1.58) | <0.001 | 1.73(1.54- 1.95) | <0.001 |
| Arthritis | 4.41(4.07-4.79) | <0.001 | 1.69(1.55- 1.85) | <0.001 |
| Cancer | 2.89(2.57-3.24) | <0.001 | - | - |
| CKD | 4.61(4.27-4.99) | <0.001 | 1.56(1.43- 1.69) | <0.001 |
| DM | 4.43(4.07-4.82) | <0.001 | 1.69(1.53- 1.86) | <0.001 |
| Dyslipidaemia | 3.27(2.92-3.67) | <0.001 | 1.85(1.62- 2.11) | <0.001 |
| Hypertension | 5.66(5.13-6.24) | <0.001 | 1.95(1.75- 2.18) | <0.001 |
| COPD | 4.41(3.87-5.03) | <0.001 | 1.44(1.25- 1.67) | <0.001 |
| Asthma | 1.56(1.41-1.72) | <0.001 | 1.54(1.36- 1.73) | <0.001 |
| CLD | 1.35(1.22-1.49) | <0.001 | 0.89(0.81- 0.99) | 0.03 |
| Obesity | 1.51(1.40-1.63) | <0.001 | 1.14(1.04- 1.25) | 0.005 |
| MetS | 2.34(2.13-2.58) | <0.001 | - | - |

ASCVD, atherosclerotic cardiovascular disease; CKD, chronic kidney disease ; DM, diabetes mellitus ; CLD, Chronic liver disease ;CHF, congestive heart failure; MetS, metabolic syndrome ;COPD: chronic obstructive pulmonary disease

OR, odds ratio; aOR, adjusted odds ratio

The results of the univariate analysis of ASCVD correlation showed that age, sex, race, education level, poverty index, smoking, and 12 chronic diseases were significantly correlated with ASCVD (all *P*<0.05). In further stepwise regression multifactor analysis, insurance, metabolic syndrome, and cancer were excluded and other variables remained statistically significant.

**eTable 6：Multivariate logistic regression with year fixed effect included**

| character | Estimate | OR | 95% CI | *P* value |
| --- | --- | --- | --- | --- |
| Sex |  |  |  |  |
| Female | ref | ref | ref | ref |
| Male | 0.58 | 1.78 | 1.78( 1.61, 1.96) | <0.0001 |
| Race/ethinicity |  |  |  |  |
| Non-Hispanic White | ref | ref | ref | ref |
| Non-Hispanic Black | -0.15 | 0.86 | 0.86( 0.78, 0.94) | 0.002 |
| Hispanic | -0.4 | 0.67 | 0.67( 0.59, 0.76) | <0.0001 |
| Other Race | 0.02 | 1.02 | 1.02( 0.83, 1.24) | 0.86 |
| Education |  |  |  |  |
| Less than high school | ref | ref | ref | ref |
| High school diploma | -0.06 | 0.94 | 0.94( 0.84, 1.06) | 0.32 |
| More than high school | -0.17 | 0.85 | 0.85( 0.75, 0.95) | 0.004 |
| Age_group |  |  |  |  |
| 20-39 | ref | ref | ref | ref |
| 40-49 | 1.04 | 2.82 | 2.82( 2.27, 3.51) | <0.0001 |
| 50-59 | 1.56 | 4.75 | 4.75( 3.89, 5.80) | <0.0001 |
| 60-69 | 2.15 | 8.62 | 8.62( 7.12,10.44) | <0.0001 |
| >=70 | 2.66 | 14.26 | 14.26(11.79,17.24) | <0.0001 |
| Ratio of family income to poverty |  |  |  |  |
| <1.3 | ref | ref | ref | ref |
| 1.3-3.49 | -0.35 | 0.71 | 0.71( 0.64, 0.78) | <0.0001 |
| 3.5-5 | -0.6 | 0.55 | 0.55( 0.49, 0.62) | <0.0001 |
| Smoke status |  |  |  |  |
| never | ref | ref | ref | ref |
| former | 0.25 | 1.29 | 1.29( 1.18, 1.41) | <0.0001 |
| now | 0.55 | 1.74 | 1.74( 1.54, 1.95) | <0.0001 |
| Year(cycle) |  |  |  |  |
| 1999-2000 | ref | ref | ref | ref |
| 2001-2002 | 0.02 | 1.02 | 1.02( 0.82, 1.27) | 0.87 |
| 2003-2004 | 0.14 | 1.15 | 1.15( 0.90, 1.47) | 0.26 |
| 2005-2006 | 0.04 | 1.04 | 1.04( 0.86, 1.25) | 0.71 |
| 2007-2008 | -0.04 | 0.96 | 0.96( 0.79, 1.17) | 0.71 |
| 2009-2010 | -0.09 | 0.92 | 0.92( 0.73, 1.14) | 0.43 |
| 2011-2012 | -0.08 | 0.92 | 0.92( 0.76, 1.12) | 0.41 |
| 2013-2014 | -0.13 | 0.88 | 0.88( 0.71, 1.09) | 0.23 |
| 2015-2016 | -0.16 | 0.85 | 0.85( 0.71, 1.03) | 0.1 |
| 2017-2018 | 0.05 | 1.05 | 1.05( 0.85, 1.30) | 0.65 |
| Arthritis |  |  |  |  |
| no | ref | ref | ref | ref |
| yes | 0.53 | 1.7 | 1.70( 1.56, 1.85) | <0.0001 |
| CKD |  |  |  |  |
| no | ref | ref | ref | ref |
| yes | 0.44 | 1.55 | 1.55( 1.43, 1.69) | <0.0001 |
| DM |  |  |  |  |
| no | ref | ref | ref | ref |
| yes | 0.53 | 1.71 | 1.71( 1.54, 1.88) | <0.0001 |
| Hyperlipidemia |  |  |  |  |
| no | ref | ref | ref | ref |
| yes | 0.62 | 1.85 | 1.85( 1.63, 2.11) | <0.0001 |
| Hypertension |  |  |  |  |
| no | ref | ref | ref | ref |
| yes | 0.67 | 1.96 | 1.96( 1.75, 2.19) | <0.0001 |
| COPD |  |  |  |  |
| no | ref | ref | ref | ref |
| yes | 0.38 | 1.47 | 1.47( 1.26, 1.70) | <0.0001 |
| Asthma |  |  |  |  |
| no | ref | ref | ref | ref |
| yes | 0.43 | 1.54 | 1.54( 1.37, 1.74) | <0.0001 |
| CLD |  |  |  |  |
| no | ref | ref | ref | ref |
| yes | -0.14 | 0.87 | 0.87( 0.79, 0.96) | 0.01 |
| Obesity |  |  |  |  |
| no | ref | ref | ref | ref |
| yes | 0.14 | 1.16 | 1.16( 1.05, 1.27) | 0.002 |

**eTable7. Multiplicative** **interactions among multimorbidity in ASCVD in a multivariable logistic regression model**

| **Variables** | **Estimate** | **Std. Error** | **aOR (95% CI)** | ***P* value** |
| --- | --- | --- | --- | --- |
| Main effects items |  |  |  |  |
| Arthritis | 0.68 | 0.14 | 1.97(1.48-2.62) | <0.001 |
| CKD | 0.73 | 0.17 | 2.07(1.48-2.89) | <0.001 |
| DM | 0.55 | 0.17 | 1.74(1.23-2.45) | 0.002 |
| Dyslipidaemia | 0.87 | 0.13 | 2.4(1.84-3.12) | <0.001 |
| Hypertension | 0.96 | 0.15 | 2.61(1.92-3.54) | <0.001 |
| COPD | 0.37 | 0.26 | 1.45(0.86-2.47) | 0.16 |
| Asthma | 0.64 | 0.18 | 1.89(1.32-2.71) | <0.001 |
| CLD | -0.27 | 0.2 | 0.77(0.51-1.14) | 0.18 |
| Obesity | 0.46 | 0.18 | 1.58(1.12-2.6) | 0.01 |
| Interaction items |  |  |  |  |
| CKD*Asthma | -0.53 | 0.13 | 0.59(0.45-0.76) | <0.001 |
| DM*Obesity | 0.26 | 0.11 | 1.29(1.03-1.62) | 0.03 |
| Hypertension*Asthma | 0.3 | 0.12 | 1.34(1.05-1.73) | 0.02 |

ASCVD, atherosclerotic cardiovascular disease; aOR, adjusted odds ratio; CI, confidence interval; CKD, chronic kidney disease ; DM, diabetes mellitus ; CLD, Chronic liver disease ; COPD, chronic obstructive pulmonary disease

Model adjusted for age, sex, race/ethnicity, education, Ratio of family income to poverty and smoking status

**eTable 8.** **Additive interactions among multimorbidity in ASCVD in a multivariable logistic regression model**

| **Model** | **Variable combination** | | **Interaction term** | **ASCVD/Total(n)** | **aOR(95% CI)** | ***P*** |
| --- | --- | --- | --- | --- | --- | --- |
| Model1 | DM | Dyslipidaemia |  |  |  |  |
|  | No | No | A0B0 | 583/14258 | ref | ref |
|  | No | Yes | A0B1 | 2580/27147 | 1.92(1.63- 2.27) | <0.001 |
|  | Yes | No | A1B0 | 210/1327 | 1.84(1.45- 2.34) | <0.001 |
|  | Yes | Yes | A1B1 | 1865/7554 | 3.22(2.73- 3.81) | <0.001 |
|  | RERI |  |  |  | 0.46(0.03-0.89) |  |
|  | AP |  |  |  | 0.14(0.01-0.28) |  |
|  | S |  |  |  | 1.26(0.93-1.71) |  |
| Model2 | Hypertension | Asthma |  |  |  |  |
|  | No | No | A0B0 | 1048/26373 | ref | ref |
|  | No | Yes | A0B1 | 209/3834 | 1.29(1.05- 1.59) | 0.02 |
|  | Yes | No | A1B0 | 3491/19142 | 1.89(1.68- 2.12) | <0.001 |
|  | Yes | Yes | A1B1 | 861/3415 | 2.84(2.42- 3.33) | <0.001 |
|  | RERI |  |  |  | 0.66(0.22-1.09) |  |
|  | AP |  |  |  | 0.23(0.1-0.36) |  |
|  | S |  |  |  | 1.56(1.05-2.3) |  |
| Model3 | Arthritis | Asthma |  |  |  |  |
|  | No | No | A0B0 | 2150/33845 | ref | ref |
|  | No | Yes | A0B1 | 341/4535 | 1.35(1.13- 1.60) | <0.001 |
|  | Yes | No | A1B0 | 2389/11670 | 1.53(1.40- 1.68) | <0.001 |
|  | Yes | Yes | A1B1 | 729/2714 | 2.31(1.94- 2.74) | <0.001 |
|  | RERI |  |  |  | 0.43(0.02-0.84) |  |
|  | AP |  |  |  | 0.19(0.03-0.34) |  |
|  | S |  |  |  | 1.49(0.97-2.29) |  |
| Model4 | CKD | Asthma |  |  |  |  |
|  | No | No | A0B0 | 2015/32620 | ref | ref |
|  | No | Yes | A0B1 | 573/5220 | 1.73(1.49- 2.00) | <0.001 |
|  | Yes | No | A1B0 | 1922/7932 | 1.71(1.55- 1.88) | <0.001 |
|  | Yes | Yes | A1B1 | 376/1300 | 1.71(1.43- 2.04) | <0.001 |
|  | RERI |  |  |  | -0.73(-1.12--0.33) |  |
|  | AP |  |  |  | -0.43(-0.71--0.15) |  |
|  | S |  |  |  | 0.49(0.32-0.75) |  |

ASCVD, atherosclerotic cardiovascular disease; aOR, adjusted odds ratio; CI, confidence interval; CKD, chronic kidney disease; DM, diabetes mellitus

Model1: Adjusted for age, sex, race/ethnicity, education, Ratio of family income to poverty, smoking status, arthritis, CKD, hypertension, COPD, asthma, CLD and obesity

Model2: Adjusted for age, sex, race/ethnicity, education, Ratio of family income to poverty, smoking status, arthritis, CKD, COPD, CLD and obesity

Model3: Adjusted for age, sex, race/ethnicity, education, Ratio of family income to poverty, smoking status, CKD, hypertension, COPD, CLD and obesity

Model4: Adjusted for age, sex, race/ethnicity, education, Ratio of family income to poverty, smoking status, arthritis, hypertension, COPD, CLD and obesity

**eFigure 11. Additive interactions between multimorbidity in ASCVD (shown in bar chart)**


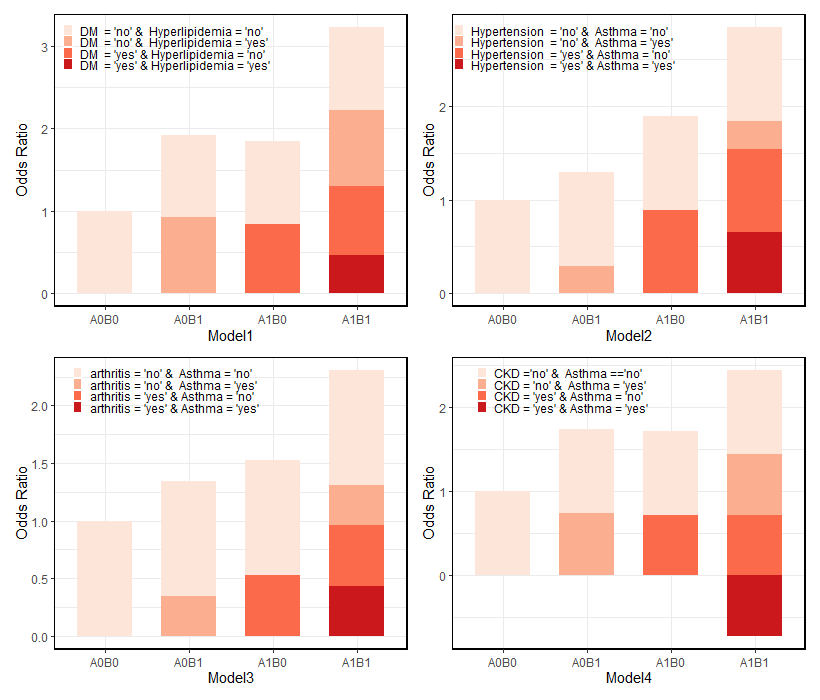


DM, diabetes mellitus; CKD, chronic kidney disease;

The meaning of A0B0, A0B1, A1B0 and A1B1 were given in the corresponding legend.

Model1: Adjusted for age, sex, race/ethnicity, education, Ratio of family income to poverty, smoking status, arthritis, CKD, hypertension, COPD, asthma, CLD and obesity

Model2: Adjusted for age, sex, race/ethnicity, education, Ratio of family income to poverty, smoking status, arthritis, CKD, COPD, CLD and obesity

Model3: Adjusted for age, sex, race/ethnicity, education, Ratio of family income to poverty, smoking status, CKD, hypertension, COPD, CLD and obesity

Model4: Adjusted for age, sex, race/ethnicity, education, Ratio of family income to poverty, smoking status, arthritis, hypertension, COPD, CLD and obesity
